# Genomic analysis reveals extensive gene duplication within the bovine TRB locus

**DOI:** 10.1186/1471-2164-10-192

**Published:** 2009-04-24

**Authors:** Timothy Connelley, Jan Aerts, Andy Law, W Ivan Morrison

**Affiliations:** 1 The Roslin Institute and Royal (Dick) School of Veterinary Studies, University of Edinburgh, Easter Bush, Roslin, EH25 9RG, UK; 2 Genome Dynamics and Evolution Group, Wellcome Trust Sanger Institute, Wellcome Trust Genome Campus, Hinxton, CB10 1SA, UK

## Abstract

**Background:**

Diverse TR and IG repertoires are generated by V(D)J somatic recombination. Genomic studies have been pivotal in cataloguing the V, D, J and C genes present in the various TR/IG loci and describing how duplication events have expanded the number of these genes. Such studies have also provided insights into the evolution of these loci and the complex mechanisms that regulate TR/IG expression. In this study we analyze the sequence of the third bovine genome assembly to characterize the germline repertoire of bovine TRB genes and compare the organization, evolution and regulatory structure of the bovine TRB locus with that of humans and mice.

**Results:**

The TRB locus in the third bovine genome assembly is distributed over 5 scaffolds, extending to ~730 Kb. The available sequence contains 134 TRBV genes, assigned to 24 subgroups, and 3 clusters of DJC genes, each comprising a single TRBD gene, 5–7 TRBJ genes and a single TRBC gene. Seventy-nine of the TRBV genes are predicted to be functional. Comparison with the human and murine TRB loci shows that the gene order, as well as the sequences of non-coding elements that regulate TRB expression, are highly conserved in the bovine. Dot-plot analyses demonstrate that expansion of the genomic TRBV repertoire has occurred via a complex and extensive series of duplications, predominantly involving DNA blocks containing multiple genes. These duplication events have resulted in massive expansion of several TRBV subgroups, most notably TRBV6, 9 and 21 which contain 40, 35 and 16 members respectively. Similarly, duplication has lead to the generation of a third DJC cluster. Analyses of cDNA data confirms the diversity of the TRBV genes and, in addition, identifies a substantial number of TRBV genes, predominantly from the larger subgroups, which are still absent from the genome assembly. The observed gene duplication within the bovine TRB locus has created a repertoire of phylogenetically diverse functional TRBV genes, which is substantially larger than that described for humans and mice.

**Conclusion:**

The analyses completed in this study reveal that, although the gene content and organization of the bovine TRB locus are broadly similar to that of humans and mice, multiple duplication events have led to a marked expansion in the number of TRB genes. Similar expansions in other ruminant TR loci suggest strong evolutionary pressures in this lineage have selected for the development of enlarged sets of TR genes that can contribute to diverse TR repertoires.

## Background

Diverse αβTR repertoires are crucial to the maintenance of effective T cell-mediated immunity [1]. Estimates based on direct measurement indicate that in humans and mice individuals express a repertoire of approximately 2 × 10^7 ^[2] and 2 × 10^6 ^[3] unique αβTRs respectively. As with the other antigen-specific receptors (IG of B cells and γδTRs of γδT cells) diversity is generated in lymphocytic precursors by somatic recombination of discontiguous variable (V), diversity (D – TRB chains but not TRA chains) and joining (J) genes to form the membrane-distal variable domains. Diversity is derived from both the different permutations of V(D)J genes used to form the TRA and TRB chains expressed by individual thymocytes (combinatorial diversity) and also by the activity of terminal deoxynucleotide transferase and exonuclease at the V(D)J junction during recombination (junctional diversity). Consequently, much of the diversity is focused in the third complementarity determining region (CDR3), which is encoded by the V(D)J junction and forms the most intimate association with the antigenic peptide component of the peptide-MHC (pMHC) ligand of αβTRs, whereas the CDR1 and CDR2 of the TRA and TRB chains, that predominantly interact with the MHC, are encoded within the germline V genes [4,5].

TRB chain genes are located in the TRB locus, which in humans is ~620 Kb long and situated on chromosome 7 and in mice is ~700 Kb and located on chromosome 6 [6-8]. In both species, the organisation of TRB genes is similar, with a library of TRBV genes positioned at the 5' end and 2 DJC clusters (each composed of a single TRBD, 6–7 TRBJ and a single TRBC gene) followed by a single TRBV gene with an inverted transcriptional orientation located at the 3'end [9,10]. The germline repertoire of TRBV genes in humans is composed of 65 genes belonging to 30 subgroups (genes with > 75% nucleotide identity), whilst in mice the repertoire comprises 35 genes belonging to 31 subgroups [10-12] The disparity between the number of TRBV genes in the 2 species is the result of multiple duplication events within the human TRB locus, most of which have involved tandem duplication of blocks of DNA (homology units) containing genes from more than one subgroup [10,13].

V(D)J recombination is initiated by site-specific DNA cleavage at recombination signal sequences (RSs) mediated by enzymes encoded by recombination activating genes (RAG) 1 and 2 [14]. RSs comprise conserved heptamer and nonamer sequences separated by spacers of either 12 bp (12-RS – located 5'to TRBD and TRBJ genes) or 23 bp (23-RS – located 3' to TRBV and TRBD genes). Correct V(D)J assembly is achieved as recombination can only occur between genes flanked with RS of dissimilar length (the '12/23 rule') and direct TRBV/TRBJ recombination is prohibited by the 'beyond 12/23' phenomenon [15-17]. As with other antigen-specific receptor loci, recombination in the TRB locus is under strict lineage-, stage- and allele-specific regulation associated with control of RAG accessibility to RSs mediated through alterations in chromatin structure (the 'accessibility hypothesis') [18-20]. Numerous studies have shown that both the TRB enhancer (Eβ) and transcriptional promoters within the TRB locus serve as RAG accessibility control elements, playing a critical role in regulating chromatin structure and therefore recombination of TRB genes [21-27].

Current knowledge of the TRB gene repertoires of agriculturally important artiodactyl species (e.g. pigs, cattle and sheep) is limited. Published analyses of rearranged TRB transcripts have demonstrated the expression of 19 TRBV subgroups in pigs [28,29], 13 subgroups in sheep [30] and 17 subgroups in cattle, some of which have undergone extensive duplication [31-34]. Information on the genomic organisation of the TRB loci is predominantly restricted to the DJC region, which in the pig was found to be composed of 2 tandemly arranged DJC clusters [35] but in sheep contained 3 tandemly arranged DJC clusters [36]. Preliminary analysis of a BAC clone corresponding to part of the DJC region indicates that in cattle the DJC region may also consist of 3 DJC clusters [37].

Sequencing of the complete TRB loci in human and mice allowed the repertoire of TRB genes in these species to be fully characterised and also permitted analysis of the organisation, regulation and evolution of this immunologically important locus [9,10]. In this study we have used the sequence of the third bovine genome assembly (Btau_3.1) to further study the bovine TRB repertoire and TRB locus. Although the sequence of the TRB locus is incomplete, the results reveal that duplication within the locus has been prolific leading to a massive expansion of TRBV gene numbers and the generation of a third DJC cluster. Furthermore, the analysis shows that the genomic organisation of the TRB locus and the non-coding elements that regulate TRB expression are highly conserved in cattle when compared to that of humans and mice.

## Results

### Extensive duplication has generated a large germline repertoire of bovine TRBV genes

A total of 134 TRBV genes, distributed over 5 scaffolds was identified in Btau_3.1 (Additional File 1). Consistent with data from fluorescent *in situ *hybridisation studies [38], the majority of the TRBV genes were located on 2 scaffolds (Chr4.003.105 [91 TRBV] and Chr4.003.108 [21 TRBV]) mapped to chromosome 4, whilst the remaining genes were located on 3 scaffolds (ChrUn.003.1717 [18 TRBV], ChrUn003.4367 [3 TRBV] and ChrUn.003.12588 [1 TRBV]) which have not been assigned a chromosomal location. Within the scaffolds are several regions of undetermined sequence, including large areas of ~35 Kb and ~147 Kb on Chr4.003.105 and Chr4.003.108 respectively.

Each TRBV gene is composed of i) a short leader (L) exon, generally of ~50 bp, ii) a single intron of between ~80 and ~500 bp and iii) a variable (V) exon of ~300 bp, immediately flanked at the 3'end with a 23-RS. Comparison of the nucleotide sequence of each of the bovine TRBV genes with human TRBV gene sequences, revealed maximum levels of similarity between the species ranging from 71.8% to 83.15% for all except one of the bovine TRBV genes. On the basis of these results, bovine TRBV genes were considered orthologues of their most similar human counterpart and were assigned to subgroups named according to the orthologous human subgroup (Table 1). The single bovine TRBV gene that lacked significant homology to any of the human TRBV genes displayed 76.6% identity with the murine TRBV1 gene (which lacks a human orthologue) and was placed in subgroup TRBVX. The subgroups thus established generally adhered to the definition of members within a subgroup exhibiting > 75% nucleotide sequence identity. However, the single member of the TRBV10 subgroup displayed > 75% identity to all of the TRBV6 genes and the identity between members of the TRBV9 and TRBV5 subgroups was often > 75% (data not shown). Conversely a single member of the TRBV19 subgroup (TRBV19f) showed only 63.0–64.8% nucleotide identity with the other members of this subgroup.

**Table 1:** TRBV gene repertoires.

Subgroup	Number of genes identified in human TRB locus		Number of genes identified in bovine TRB locus		Number of bovine genes identified from cDNA analyses
	Total	Functional	Total	Functional	

1	1				
2	1	1			
3	1–2*	1	1	1	1
4	2–3*	2–3	2	2	2
5	8	5	4	1	1
6	8–9*	6–7 (+1)†	40	20	20
7	9	5 (+2)†	2	2	3
8	2				
9	1	1	35	23	13
10	3	2 (+1)†	1	0	2
11	3	3	1	0	
12	5	3	2	2	2
13	1	1	1	0	
14	1	1	1	1	1
15	1	1	1	1	1
16	1	(+1)†	1	0	1
17	1				
18	1	1	4	0	
19	1	1	6	3	3
20	1	1	5	5	9
21	1		16	9	11
22	1				
23	1				
24	1	1	1	1	2
25	1	1	1	1	1
26	1		1	1	1
27	1	1			1
28	1	1	1	1	1
29	1	1	5	3	8
30	1	(+1) †	1	1	1
X			1	1	1

Total	64–67	40–42 (+6)	134	79	86

The total and functional repertoire of TRBV genes in the human TRB locus and in the third assembly of the bovine genome as well as the bovine TRBV gene repertoire identified from cDNA analysis. Details of the human repertoire are taken from the IMGT database . *denotes variation in germline repertoire due to insertion/deletion polymorphisms and † denotes TRBV genes which have alternative functional and non-functional alleles (TRBV6-2, TRBV7-3, TRBV7-4, TRBV10-1, TRBV16, TRBV30).

**Figure 1:**
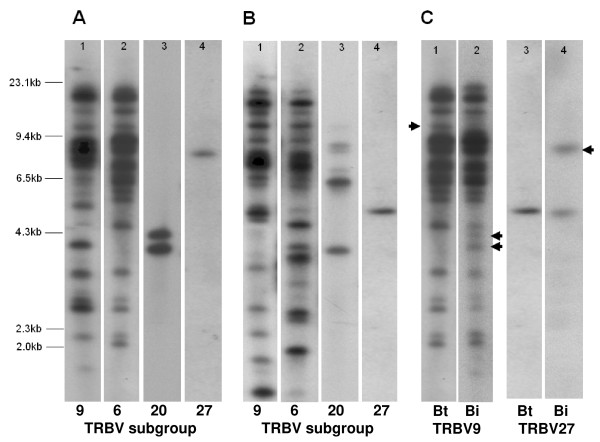
**Southern blot analysis of bovine genomic DNA**. Genomic DNA from a Bos taurus animal digested with (A) HindIII or (B) Ssp1 was hybridised with probes specific for TRBV9 (lane 1), TRBV6 (lane 2), TRBV20 (lane 3) and TRBV27 (lane 4). (C) Comparison of the banding patters obtained from genomic DNA of a *Bos taurus *(Bt) and a *Bos indicus *(Bi) animal hybridised with a probe specific for TRBV9 (lanes 1 and 2) after digestion with HindIII and a probe specific for TRBV27 after digestion with Ssp1 (lanes 3 and 4). Arrows indicate bands that are evident in *Bos taurus *but not *Bos indicus *DNA or *vice versa*.

Of the 24 bovine subgroups present in the genome assembly, 11 have multiple members. Subgroups TRBV6, 9 and 21 have all undergone substantial expansion, having 40, 35 and 16 members respectively – together representing 68% of the total Btau_3.1 TRBV gene repertoire. Southern blot analysis corroborates the presence of large numbers of TRBV6 and 9 genes in the genome (Figure 1).

**Figure 2:**
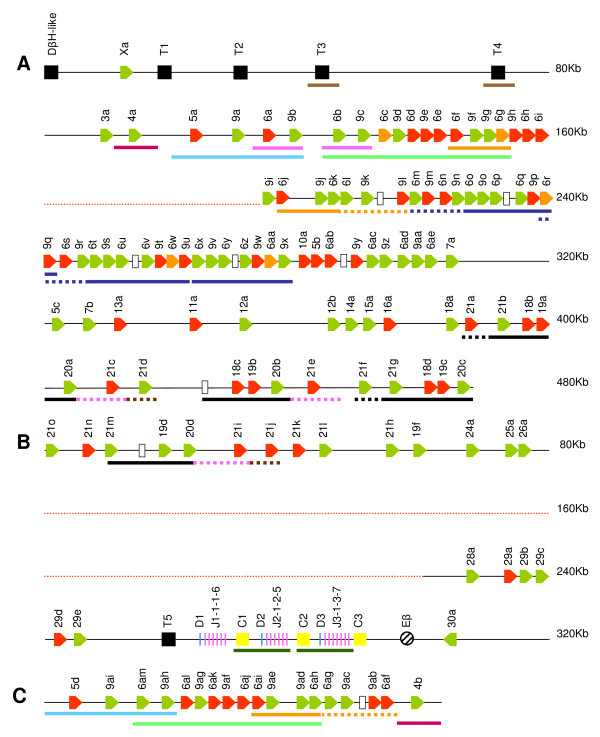
**Genomic organisation of the genes in the bovine TRB locus as described in Btau_3.1**. The order and location of TRB genes on (A) Chr4.003.105 (B) Chr.4.003.108_RC and (C) ChrUn.003.1717. Red dotted lines represent large regions of undetermined sequence within the scaffolds. TRBV genes are classified as functional (green), open-reading frame non-functional (orange) or pseudogenes (red), and their transcriptional orientation indicated by their direction; TRBV gene 'relics' are shown as open boxes. TRBD (blue vertical lines), TRBJ (pink vertical lines) and TRBC (yellow boxes) genes are arranged into 3 DJC clusters, with a putative bovine TRB enhancer (Eβ) located 3' to the TRBC3 gene (black diagonal shading). The sizes of non TRB genes (black boxes) – dopamine-β-hydroxylase-like gene (DβH-like) and trypsinogen genes (T) are not shown to scale. Regions of duplicated DNA are indicated by the colour-coordinated boxes located beneath the scheme of gene location. Broken lines indicate regions of DNA that are not present in all copies of the duplicated region.

**Figure 3:**
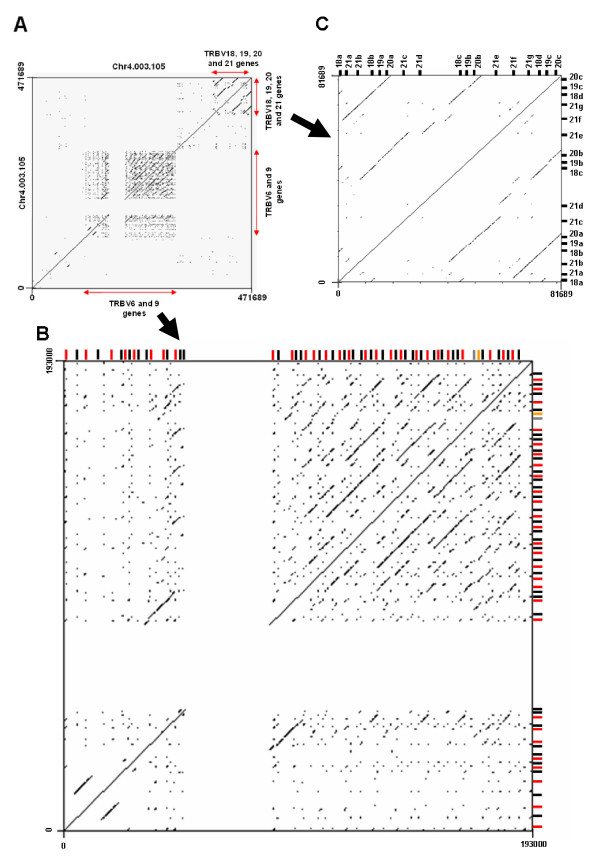
**Dot-plot analyses of Chr4.003.105**. (A) The TRB locus region of Chr4.003.105. The multiplicity of diagonal lines parallel to the main diagonal present in the regions containing i) the TRBV 6 and 9 genes and ii) the TRBV 18, 19, 20 and 21 genes shows that these regions have been subjected to numerous duplication events. The clear cruciform area in the TRBV 6 and 9 region (also in (B)) reflects an 35 Kb area of undetermined sequence. (B) The TRBV 6 and 9 region of Chr4.003.105. Various duplicated regions of ~7 Kb to ~31 Kb and including multiple TRBV6 (black) and TRBV9 (red) genes are evident. (C) The TRBV18, 19, 20 and 21 region of Chr4.003.105. The pattern of parallel lines in this dot-plot analysis indicates a region of DNA that includes a TRBV21, 18, 19 and 20 genes that has been duplicated twice, giving rise to 3 homology units.

A prominent feature of the genomic organisation of TRBV genes (Figure 2) is that members of expanded subgroups are generally intercalated with members of other expanded subgroups in a recurrent pattern. Thus, a 165 KB region of Chr4.003.105 and virtually all of scaffold ChrUn.003.1717 are composed of alternating TRBV6 and 9 genes (reflected in the similarity in the patterns of larger bands (> 4.3 Kb) obtained in southern blots of genomic DNA when hybridised with TRBV9- and TRBV6-specific probes in Figure 1), whilst the 3'end of Chr4.003.105 and the 5' end of Chr4.003.108 contain repeated units comprising TRBV18, 19, 20 and 21 genes. Dot-plot analyses indicate that this organisation has arisen through a series of complex tandem duplication events within the regions in which TRBV9 and 6 genes and TRBV18, 19, 20 and 21 genes are located (Figure 3). Six homology units, ranging in size from ~7 Kb to ~31 Kb and encompassing from 1 to 11 TRBV genes were identified. Three of these homology units (represented by the orange, dark blue and black bars in Figure 2) have undergone multiple (2–3) duplications: variation in the length of the different copies of these homology units (represented by broken lines in Figure 2), suggests that either i) distinct iterations of a duplication event have involved different components of the homology unit or ii) the different copies have been subject to different post-duplication deletions.

The levels of nucleotide identity between TRBV genes in corresponding positions in homology units is frequently high: 12 pairs of TRBV6 genes, 11 pairs of TRBV9 and 1 pair each of TRBV19 and TRBV20 have identical coding sequences whilst 1 pair of TRBV4 genes and 3 pairs of TRBV21 as well as 4 triplets of TRBV6 and 4 triplets of TRBV9 genes have > 97% sequence identity in the coding region.

### Duplication has expanded the repertoire of TRBD, TRBJ and TRBC genes in the bovine genome

**Figure 4:**
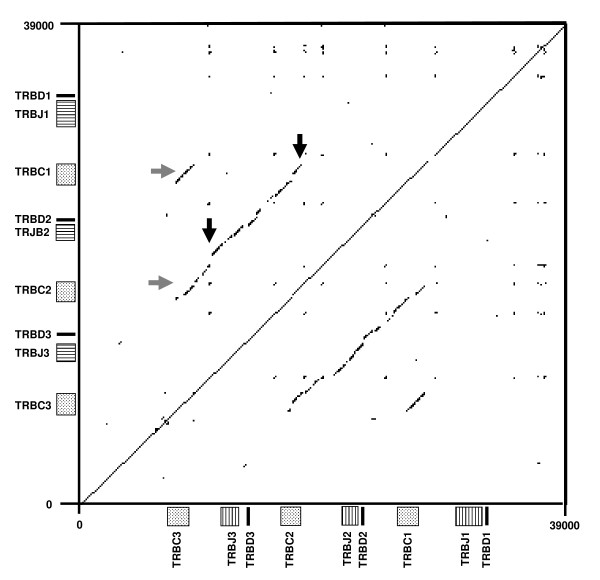
**Dot-plot analysis of the bovine DJC region on Chr4.003.108**. Duplication of a ~7 Kb region (diagonal line between black arrows) has generated a third DJC cluster. One of the homology units incorporates TRBC1, TRBD2 and the TRBJ2 whilst the other incorporates TRBC2, TRBD3 and TRBJ3. Smaller lines parallel to the main diagonal reflect the similarity in sequence of TRBC3 with TRBC1 and 2 (grey arrows).

A total of 3 TRBD, 18 TRBJ and 3 TRBC genes were identified in the assembly (Additional File 1). These genes were all located within a ~26 Kb region of scaffold Chr4.003.108 and organised into 3 tandemly arranged clusters, each of ~7 Kb length and composed of a single TRBD gene, 5-7 TRBJ genes and a single TRBC gene (Figure 2). Dot-plot analysis reveals the presence of a third DJC cluster is attributable to duplication of a ~7 Kb region, one copy of which incorporates TRBC1, TRBD2 and the TRBJ2 cluster whilst the other copy incorporates TRBC2, TRBD3 and the TRBJ3 cluster (Figure 4). Numerous interruptions in the line representing the duplicated region indicate that there has been significant post-duplication deletion/insertion related modification of the duplicated region.

**Figure 5:**
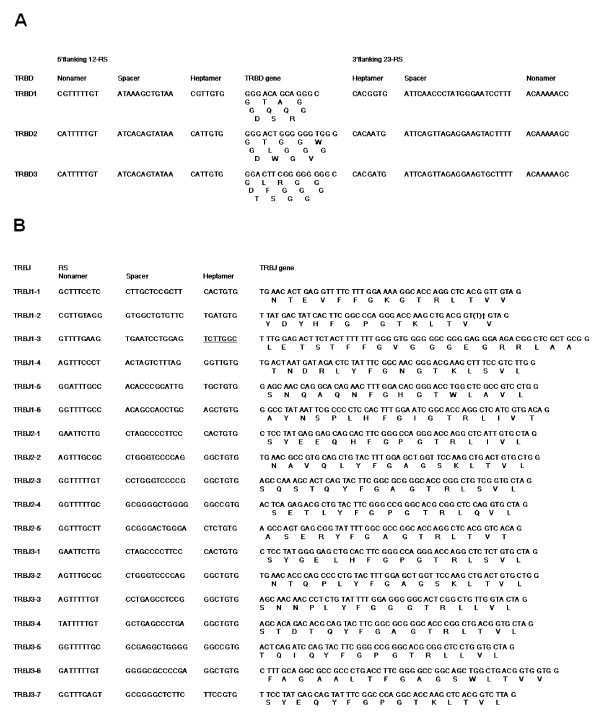
**The genomic sequence of the (A) 3 TRBD genes and (B) 18 TRBJ genes**. The nucleotide and predicted amino acid sequences of (A) The TRBD genes. TRBD genes have the potential to be read in all 3 reading frames, and with the exception of the 3^rd ^reading frame of TRBD1 encode at least 1 glycine residue. (B) The TRBJ genes. TRBJ1-3 is predicted to be non-functional due to loss of consensual RS heptamer sequence (bold and underlined).(†) In the genome TRBJ1-2 has a frameshift due a single base pair deletion in the TRBJ region and would therefore be predicted to be a pseudogene, but based on sequences correlating with this TRBJ gene derived from cDNA analyses we have introduced a thymidine (shown in parentheses).

The nucleotide and deduced amino acid sequence of the 3 TRBD and 18 TRBJ genes as well as the flanking RS are shown in Figure 5a and 5b respectively. The 13 bp (TRBD1) or 16 bp (TRBD2 and 3) TRBD genes are G-rich and encode at least one glycine in all 3 potential reading frames with the exception of the 3^rd ^reading frame of TRBD1. The TRBJ genes range in size from 43 bp to 59 bp in length and all encode the canonical FGXG amino acid motif that defines TRBJ genes.

**Figure 6:**
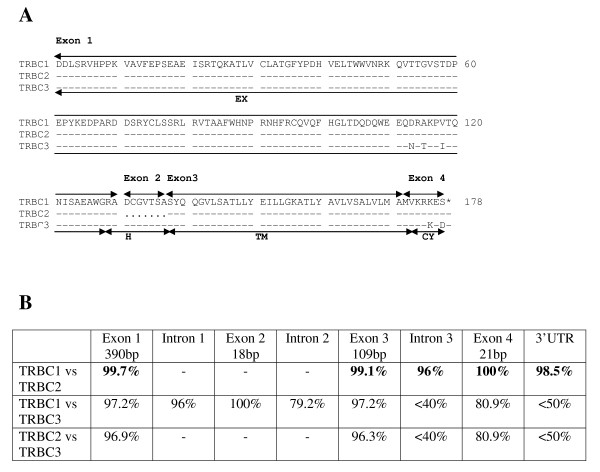
**The bovine TRBC genes**. (A) Predicted amino acid sequences of products of the TRBC1, 2 and 3 genes. The regions encoded by the 4 exons and the boundaries between the predicted extra-cellular (EX), hinge (H), trans-membrane (TM) and cytoplasmic (CY) domains have been marked. The sequence for a 7 amino acid section of TRBC2 can not be predicted due to absence of nucleotide sequence for exon 2 (represented by dots). (B) Pairwise percentage identity of nucleotide sequences between the exons, introns and 3'UTR of the 3 TRBC genes. Some pairwise comparisons have been omitted due to a region of undetermined sequence spanning the 1^st ^intron, 2^nd ^exon and 2^nd ^intron of TRBC2.

**Figure 7:**
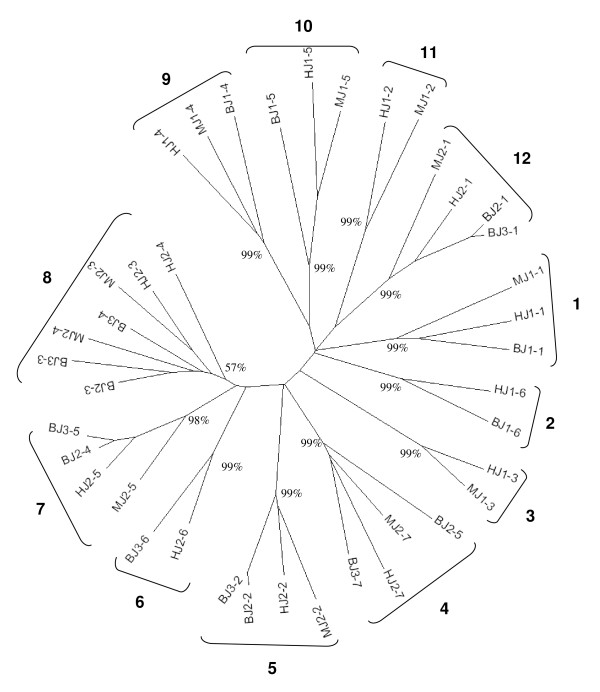
**Neighbor-joining phylogenetic tree of the functional genomic repertoire of murine, human and bovine TRBJ genes**. Analysis was completed on the coding and RS nucleotide sequence of functional TRBJ genes following complete deletion to remove gaps in the alignment. The final dataset included 59 positions. The 12 phylogenetic groups (1–12) have been indicated and the percentage bootstrap interior branch test value (P_B_) based on a 1000 replications is shown for each of the groups. Generally each group is composed of genes from the 3 species that share a conserved order in the genome; group 8 is unique in containing the orthologues of two adjacent genes human and murine TRBJ2-3 and TRBJ2-4 (and in the bovine TRBJ3-3 and TRBJ3-4 as well as TRBJ2-3).

As with all mammalian TRBC genes so far characterised, bovine TRBC1 and TRBC3 genes are composed of 4 exons, 3 introns and a 3'UTR region. The structure of the TRBC2 gene is anticipated to be the same but due to a region of undetermined sequence between exons 1 and 3 we were unable to identify exon 2. The exon nucleotide sequences of TRBC1 and 3 are very similar (97%), resulting in the encoded 178 amino acid products differing by only 5 residues – 3 in the extra-cellular domain and 2 in the cytoplasmic domain (Figure 6a). The incomplete sequence for TRBC2 is predicted to encode a product identical to that of TRBC1. In contrast to the high levels of pairwise identity between the exonic nucleotide sequences of all 3 TRBC genes, the nucleotide sequences of the 3^rd ^intron and the 3'UTR regions of TRBC3 show low identity with TRBC1 and 2, whereas the latter two genes show a high level of identity (Figure 6b). The similarity in the lengths of TRBD2 and 3, the phylogenetic clustering of TRBJ2 and TRBJ3 genes in corresponding genomic positions (Figure 7) and the similarity in the sequences of the 3^rd ^introns and 3'UTRs of TRBC1 and 2 all reflect the duplication history of the DJC region as described in Figure 4.

### The repertoire of functional TRBV, TRBD and TRBJ genes available for somatic recombination is large and phylogenetically diverse

Computational analysis was used to predict the functional competency of the TRBV, TRBD and TRBJ genes present in the genome assembly. Fifty-five (41%) of the TRBV genes identified are predicted to encode pseudogenes (Additional File 2), whilst TRBJ1-2 (which has a 1 bp deletion that results in the canonical FGXG motif being lost in the ORF) and TRBJ1-3 (which lacks a RS that is compatible with somatic recombination) are also predicted to be non-functional (Figure 5). Thus, the functional repertoire comprises 79 (59%) TRBV genes (comprising 66 unique coding TRBV sequences) belonging to 19 different subgroups, 3 TRBD genes and 16 TRBJ genes. This provides a potential 3168 (66 × 3 × 16) unique VDJ permutations that can be used during somatic recombination of TRB chains.

**Figure 8:**
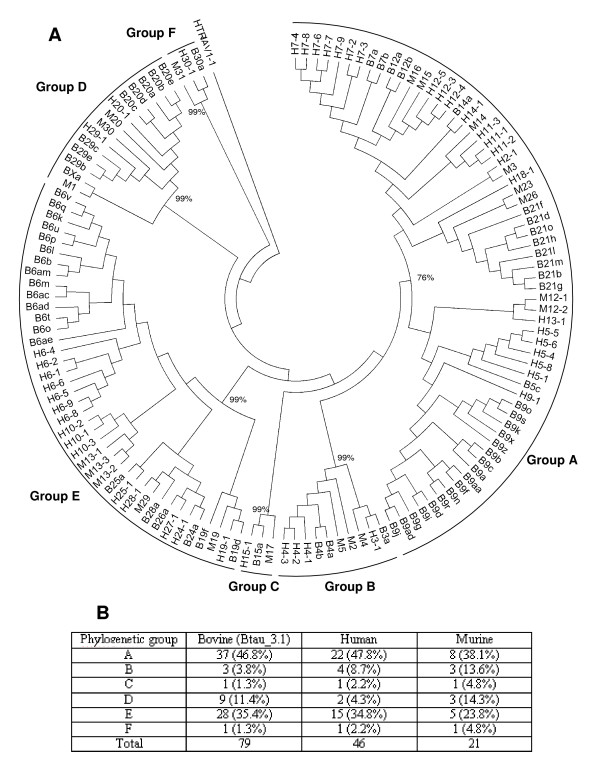
**Neighbour-joining phylogenetic tree of the functional genomic repertoire of murine, human and bovine TRBV genes**. (A) Analysis of the coding region nucleotide sequences of functional TRBV genes following complete deletion to remove gaps in the alignment. The final dataset included 281 positions. The sequence of HTRAV1-1[93] has been included as an outgroup. The six phylogenetic groups (A-F) have been indicated and the percentage bootstrap interior branch test value (P_B_) based on a 1000 replications is shown for each of the groups. To reduce the size of the tree for presentation, where 2 TRBV genes have identical sequence only 1 has been included in the analysis. Therefore, bovine 6x (identical to 6t), 6an (6p), 6u (6y), 6ag (6l), 6ak (6h), 6v (6z), 9ae (9f), 9ak (9b), 9ac(9k), 9s (9v), 9aj (9d), 19d (19e) and 21m (21p) have been excluded, as has human TRBV6-3 which is identical to TRBV6-2. H = human, M = murine, B = bovine. (B) Summary of the distribution of functional TRBV genes amongst the 6 phylogenetic groups in humans, mice and Btau_3.1.

Phylogenetic analysis demonstrates that the repertoire of functional TRBV genes is diverse (Figure 8), with representatives in each of the 6 phylogenetic groups (A-F) described previously in humans and mice [13,39]. The phylogenetic groupings were supported by high (99%), bootstrap values (P_B_), with the exception of group A (P_B _= 76%). Maximum likelihood analysis using a variety of nucleotide models provides essentially similar phylogenetic clustering (data not shown) indicating the reliability of the tree presented in Figure 8. The extensive intermingling of murine, human and bovine TRBV subgroups is consistent with the establishment of distinct subgroups having occurred prior to mammalian radiation. Conversely, the formation of distinct clades of TRBV genes of orthologous subgroups from different species (e.g. TRBV6 genes from human and bovine form distinct clades) indicates that duplication within subgroups has predominantly occurred post-speciation. Despite this and the substantial disparity in the number of functional TRBV genes present in the 3 species, the distribution amongst the different phylogenetic groups is similar (Figure 8b). Phylogenetic groups C and F form a minor component of the functional TRBV repertoire, whilst the contributions from groups B and D are also fairly modest. In contrast, group E and to an even greater extent group A, are over-represented, together representing between 61.9% (in the mouse) and 81.6% (in humans) of the total functional repertoire.

Phylogenetic analysis resolves the functional TRBJ genes in human, mice and Btau_3.1 into 12 groups (Figure 7). With the exception of group 8, each group is supported by high P_B _values and is composed of orthologues that share a conserved order in the genome; consistent with the duplication history of the DJC region, TRBJ genes from both the 2^nd ^and 3^rd ^bovine DJC clusters group together with the respective genes from the 2^nd ^murine and human DJC clusters. Group 8, which contains TRBJ2-3, human and murine TRBJ2-4 and bovine TRBJ3-3 and 3–4 genes is only supported by a P_B _value of 57%. The diversity of the functional TRBJ repertoire across the 3 species is comparable, with humans having functional genes in each of the 12 phylogenetic groups whilst in both mice and Btau_3.1 only 2 groups lack functional members: groups 3 (TRBJ1-3) and 11 (TRBJ1-2) in Btau_3.1 and groups 2 (TRBJ1-6) and 6 (TRBJ2-6) in mice.

### Comparison with cDNA data identifies additional TRBV gene sequences missing from the genome assembly

Using a variety of RT-PCR based methods, our group has isolated and sequenced over 1000 partial TRB chain cDNAs [31-33,40]. With a few exceptions, these cDNA sequences incorporated > 230 bp of the TRBV gene (i.e. over 80% of the sequence encoding the mature peptide) and in some cases the full length of the TRBV gene. Based on the assumption that sequences sharing ≤ 97% nucleotide identity represent distinct genes, as applied in studies of human and murine TRBV genes [41,42], our analysis identified 86 putative unique TRBV genes belonging to 22 subgroups (Table 1). Analysis of the sequence data available for each cDNA sequence indicated that only one of these genes is predicted to be non-functional (TRBV6-6 – due to a loss of a conserved cysteine encoding codon at position 104 according to the IMGT numbering system [43]), consistent with evidence that mRNA expression of non-functional TRB chains is down-regulated and therefore limited [10,44,45].

All of the TRBV subgroups identified in Btau_3.1 were also identified in the cDNA sequences. In addition a single member of subgroup TRBV27, which is not represented in Btau_3.1, was identified. Although the repertoire of cDNA and functional genomic TRBV genes is broadly similar in both size and distribution across the subgroups (Table 1), detailed comparison shows that for the large subgroups there is substantial disparity between the genes present in the assembled genome and the cDNA repertoire. Thus, only 23 (35.9%) of the 64 TRBV genes in subgroups TRBV6, 9, 19, 20, 21 and 29 identified from cDNA analysis had genomic sequences showing 100% sequence identity, whilst 26 (40.6%) exhibited ≤ 97% identity to any genomic sequence. The remaining 15 (23.4%) sequences displayed nucleotide identities of 98–99% with genomic gene sequences. Given the presence in the genome of TRBV genes exhibiting > 97% nucleotide identity, it is not possible to conclude whether these cDNAs represent allelic variants of already identified genes or products of additional genes absent from the current assembly. That at least some of the cDNAs fall into the latter category, is supported by the identification of sequences exhibiting 100% identity with 4 of these cDNA sequences, in the genome project's WGS trace archive (data not shown). Conversely, 40 (63.5%) of the 63 predicted functional genes identified in these subgroups within the genome did not have cDNA sequences displaying 100% nucleotide identity. Twenty-two of these (34.9%) showed 98–99% identity with cDNA sequences, whilst the remaining 18 (28.6%) exhibited < 97% identity to any of the cDNA sequences. In contrast to the findings with multi-member subgroups, cDNAs corresponding to 9 subgroups with single members identified in the genome showed 100% identity with their respective genome sequence. Thus, comparison with cDNA evidence suggests that substantial numbers of genes, predominantly from the large subgroups, are still absent from Btau_3.1.

In contrast to the TRBV situation, all TRBD and TRBC genes and the 16 functional TRBJ genes identified in Btau_3.1 have been found expressed in cDNA. In addition, a functional allele of the TRBJ1-2 gene, which compared to the genomic sequence has a 1 bp insertion that restores the ORF encoding the FGXG motif (Figure 5), has been identified. No evidence for further TRBD, TRBJ or TRBC genes was found from cDNA analysis, suggesting the repertoire of these genes in Btau_3.1 is complete.

### Conserved synteny between the human TRB locus and scaffolds Chr4.003.105 and Chr4.003.108

**Figure 9:**
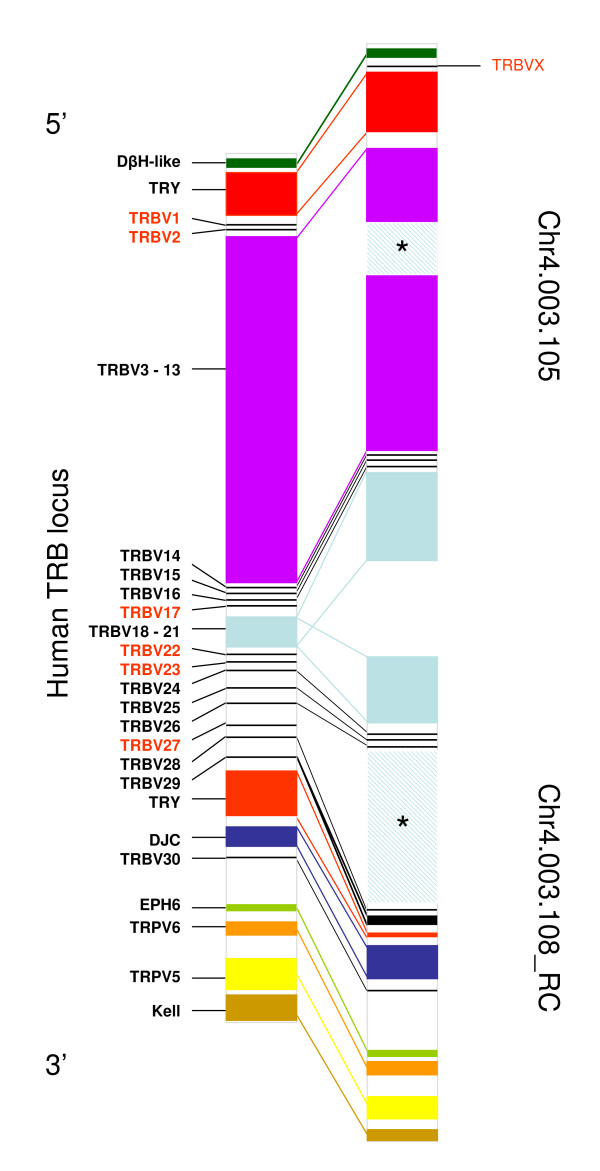
**Comparison of the genomic organisation of genes on Chr4.003.105 and Chr4.003.108 with the human TRB locus**. The relative position of genes or groups of genes in the human TRB locus and the orthologues on Chr4.003.105 and Chr4.003.108 are shown. Human TRBV genes without bovine orthologues are shown in red script, as is bovine TRBVX which lacks a human orthologue. The hatch areas marked with an asterisk in Chr4.003.105 and Chr4.003.108 indicate large areas of undetermined sequence. DβH-like (dopamine-β-hydroxylase like gene), TRY (trypsinogen genes), EPH-6 (ephrin type-b receptor 6 precursor), TRPV5 (transient receptor potential cation channel subfamily V member 5), TRPV6 and Kell (Kell blood group glycoprotein).

The organisation of genes within Chr4.003.105 and Chr4.003.108 shows marked conservation in order with that of genes at the 5' and 3'ends of the human TRB locus respectively (Figure 9). Genes belonging to orthologous TRBV subgroups show a similar order although in some areas substantial tandem duplication has obscured synteny at the level of individual genes (e.g. the TRBV3-13 regions in the human TRB locus and on Chr4.003.105). TRBVX, the only bovine TRBV gene that has no human orthologue, is located in a position (between the dopamine-β-hydroxylase-like (DβH-like) gene and trypsinogen genes) syntenic with its murine orthologue (mTRBV1). As mentioned previously, synteny is also shown in the organisation of TRBJ genes, with human and bovine orthologues occupying conserved positions in their relative clusters.

Synteny also extends to non-TRB genes located within and adjacent to the human TRB locus. The 5 trypsinogen genes located on Chr4.003.105 and Chr4.003.108 are syntenic to those located towards the 5'end and 3'end of the human TRB locus respectively, and the DβH-like gene flanking the 5' end of the human TRB locus and the ephrin type-b receptor 6 precursor (EPH6), transient receptor potential cation channel subfamily V (TRPV) member 5, TRPV6 and kell blood group glycoprotein (kell) loci flanking the 3'end of the human TRB locus all have bovine orthologues in syntenic positions on the 2 scaffolds.

Although fluorescent *in situ *hybridisation studies have previously shown that the position of the TRB locus with respect to the blue cone pigment (BCP) and chloride channel protein 1 (CLCN1) genes are conserved between ruminants and humans [46], this analysis shows for the first time the high levels of synteny between human and bovine orthologues both within and adjacent to the TRB locus. Extrapolation of this synteny predicts that Chr4.003.105 and Chr4.003.108 (in reverse complement) should be juxtaposed on chromosome 4, whilst Chr4.003.106, which contains bovine orthologues to numerous genes that in humans are telomeric to the TRB locus (including CLCN1) should be located 3' to Chr4.003.108 and Chr4.003.107, which contains a bovine orthologue to the acylglycerol kinase (AGK) gene that in humans lies centromeric to the TRB locus, should be positioned 5' to Chr4.003.105. This location of Chr4.003.106 has also been predicted by clone paired-end analysis (data not shown).

### RS and regulatory elements sequences are conserved in the bovine TRB locus

**Figure 10:**
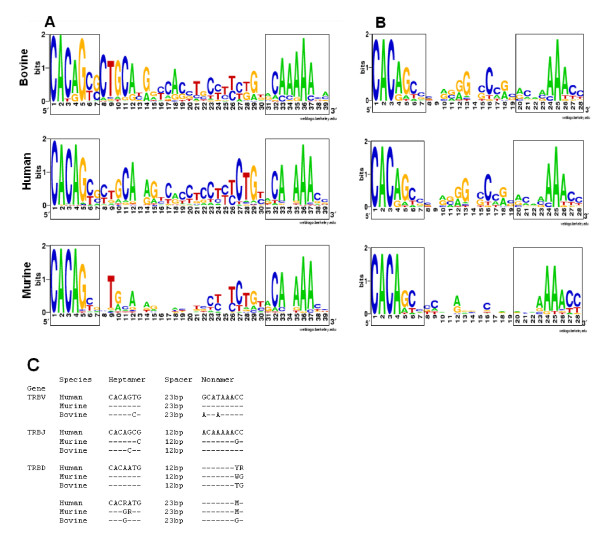
**Comparison of recombination signal sequences of human, murine and bovine TRB genes**. Sequence logos for the RSs of (A) TRBV and (B) TRBJ genes of bovine, humans and mice. The heptamer and nonamer sequences are enclosed in boxes. The height of the letters is correlated to their conservation at each location. (C) Consensus sequences of RSs from the different groups of bovine, human and murine TRB genes. Dashes represent nucleotide identity. M = A/C, R = A/G, W = A/T, and Y = C/T. Non-functional RSs functional have been excluded from the analysis.

The RS sequences of the bovine TRBV, TRBJ and TRBD genes show a high degree of similarity to canonical RS sequences defined for the corresponding human and murine genes (Figure 10). In the bovine TRBV 23-RS sequences the CACAG of the heptamer and a poly-A stretch in the centre of the nonamer show a high degree of intra- and inter-species conservation. Although conservation of the spacer is less marked, the CTGCA sequence proximal to the heptamer is reasonably well conserved and similar to that of humans. Despite more limited conservation, the 8 bp proximal to the nonamer also displays a degree of cross-species similarity. Similarly, the bovine TRBJ RS exhibits intra- and inter-species conservation of the first 3 bp (CAC) of the heptamer sequence and a poly-A stretch in the nonamer. Conservation in the spacer is limited but overrepresentation of G at the position 6 bp from the heptamer and C 4 bp from the nonamer is seen in both the bovine and human.

**Figure 11:**
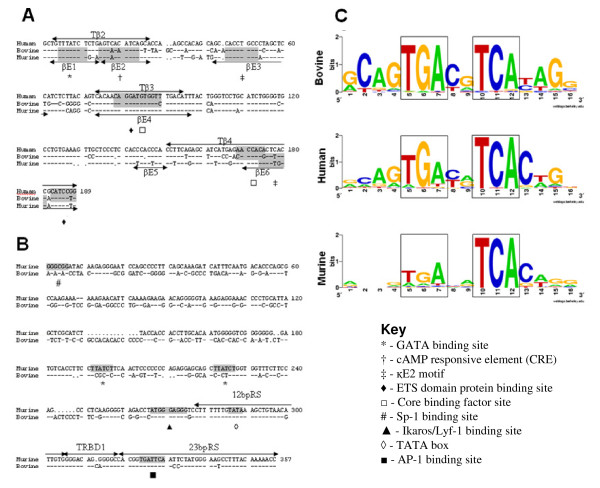
**Sequence comparison of regulatory elements in the bovine, human and murine TRB loci**. (A) Alignment of the human, bovine and murine minimal enhancer sequences. Protein binding sequences in the human (Tβ) and murine (βE) minimal Eβ region are indicated by arrows. (B) Alignment of the murine PDβ1 with the bovine sequence immediately upstream of the TRBD1 gene. The position of the TRBD1 gene and the flanking RSs are indicated. Shaded areas indicate the location of defined transcription regulation elements as defined in the key. Identity is shown by dashes and gaps by dots. (C) Sequence logos for the conserved TGAxxTCA CRE motif and adjacent bases found in the promoter regions (at ~80–120 bp upstream) of 57 bovine, 52 human and 23 murine TRBV genes. The location of conserved TGA and TCA are enclosed in boxes. The height of the letters is correlated to their conservation at each location.

We identified a 187 bp sequence ~8.7 Kb 3' to the TRBC3 gene that displays high nucleotide similarity with the sequences of the enhancers (Eβ) identified in the murine (76.2%) and human (78.3%) TRB loci [47-49]. Sequences of the protein binding sites described in the Eβs of humans (Tβ2-4) and mice (βE1-6) are well conserved in the aligned bovine sequence (Figure 11a); several of the transcription binding sites shown to be functionally important in the regulation in Eβ function [47-50], such as the GATA-binding site in βE1/Tβ2 and the κE2-binding motif in βE3 are absolutely conserved, whilst others (such as he CRE in βE2/Tβ2) show minimal sequence divergence. In contrast, the sequence of the TRBD1 promoter (PDβ1), which includes the ~300 bp directly upstream of the TRBD1 gene and has been well defined in the mouse [51,52], displays a more limited nucleotide identity (59.2%) with the bovine sequence. As shown in Figure 11b, some transcription factor binding sites demonstrated to be important for PDβ1 function (SP-1 and GATA) in mice and/or humans are absent from the bovine sequence, whereas others (TATA box, AP-1 and Ikaros/Lyf-1) have been well conserved [51-53].

We were also able to identify a conserved cAMP responsive element (CRE) motif (AGTGAxxTGA) in the ~80–120 bp upstream sequence of 57 (42.6%) of the bovine TRBV genes (Figure 11c). This motif is found within conserved decamer sequences in the promoter regions of some murine and human TRBV genes [54] and has been shown to specifically bind a splice variant of a CRE binding protein preferentially expressed in the thymus [55]. In general, the CRE motif has been found in bovine genes that are members of subgroups that are orthologous to the human TRBV subgroups in which the CRE motif is also found [10].

## Discussion

Sequencing of the human and murine TRB loci has defined the repertoire of TRB genes in these species as well as provided insights into the organisation, evolution and regulation of this immunologically important locus [9,10]. Although the bovine TRB locus sequence in the third bovine genome assembly is incomplete, the analysis conducted in the present study has provided insight into the nature of the bovine TRB gene repertoire and its genomic organisation and evolution.

The most striking result from the study was the large number of TRBV genes identified (134) which is over twice the number found in humans and four times that in mice [11,12]. Although 11 of the 24 bovine subgroups identified in the genome contain multiple genes, the majority of the TRBV repertoire expansion is attributable to the extensive membership of just 3 subgroups, TRBV6 (40 members), 9 (35 members) and 21 (16 members). By comparison, the largest subgroups in humans are TRBV6 and TRBV7, with 9 members each, whilst in mice the only multi-membered subgroups are TRBV12 and 13 with 3 members each. As in humans the expansion of the TRBV repertoire has predominantly occurred through the tandem duplication of DNA blocks containing genes from more than 1 subgroup [9,10]. Dot-plot analyses shows that this duplication in the bovine is complex, leading to the generation of 6 homology units ranging in size from ~7 Kb to ~31 Kb and encompassing between 1 and 11 TRBV genes. Unequal cross-over (non-homologous meiotic recombination) between genome-wide repeats (e.g. SINEs, LINEs and LTRs) has been proposed to act as the substrate for such duplication events in TR loci [9]. Although genome-wide repeats are found in the DNA surrounding the bovine TRBV genes (Additional file 3), as in the human TRB locus they are only rarely found at the boundaries of duplicated homology units (data not shown), suggesting their contribution to mediating duplication is minimal [10].

Although gene conversion of TRBV genes has been documented [56], as with other multi-gene families in the immune system, TRBV genes predominantly follow a 'birth-and-death' model of evolution [13,57,58], by which new genes are created by repeated gene duplication, some of which are maintained in the genome whilst others are deleted or become non-functional due to mutation. Genes maintained following duplication are subject to progressive divergence, providing the opportunity for diversification of the gene repertoire. Gene duplication within the TR loci has occurred sporadically over hundreds of millions of years with ancient duplications accounting for the generation of different subgroups and more recent duplications giving rise to the different members within subgroups [9,59]. The continuous nature of duplication and divergence of bovine TRBV genes is evident in the multi-membered subgroups where nucleotide identity between members ranges between 75.5% and 100%. The complete identity observed between some TRBV genes suggests that some of the duplication events have occurred very recently. Similar features have been described for the murine TRA and human IGκ loci, within which recent duplications, < 8 million years ago (MYA), have created pairs of V genes exhibiting ~97% nucleotide identity [9,60,61]. Southern blot data showing differences in RFLP banding patterns of TRBV9 and 27 genes in DNA from *Bos indicus *and *Bos taurus *animals (Figure 1c), which only diverged between 0.25 – 2 million years ago [62-64], provides further evidence of recent evolutionary development of the TRBV repertoire in cattle.

The distribution of TRBV genes over 5 scaffolds and the presence of > 180 Kb of undetermined sequence within two of the scaffolds indicate that characterisation of the genomic TRBV repertoire remains incomplete. Comparison with cDNA sequences data indicates that the number of undefined genes is substantial – only 36/86 (42%) of TRBV genes identified from cDNA analysis have corresponding identical sequences in Btau_3.1. Most of the identified TRBV genes missing from the assembly are members of the large subgroups TRBV6, 9, 19, 20, 21 and 29, further enhancing their numerical dominance. Although it is anticipated that completion of the TRB locus sequence will incorporate significant numbers of additional TRBV genes, the possible existence of insertion-deletion related polymorphisms (IDRPs), which can lead to intra-species variation in genomic TRBV gene repertoires as described in human and murine TRB loci [65-68], may result in some of the genes identified in cDNA being genuinely absent from the sequenced bovine genome

The proportion of TRBV pseudogenes in Btau_3.1 is 41%, comparable to that seen in both humans (29%) and mice (40%), suggesting that the 'death rate' in TRBV gene evolution is generally high [58]. Pseudogene formation has occurred sporadically throughout the evolution of TRBV genes, with genes that have lost function tending to subsequently accumulate further lesions [9]. The majority of bovine TRBV pseudogenes (57%) contain a single lesion and thus appear to have arisen recently; the remaining 43% have multiple lesions of varying severity and complexity (Additional file 2). In addition to pseudogenes we also identified 7 sequences showing limited local similarity to TRBV genes in Btau_3.1 (Figure 2 – open boxes). Such severely mutated TRBV 'relics', 22 of which have been identified in the human TRB locus [10]., are considered to represent the remnants of ancient pseudogene formation.

In contradiction to a previous report [39], the repertoire of functional TRBV genes in Btau_3.1 exhibits a level of phylogenetic diversity similar to that of humans and mice. Phylogenetic groups A and E are over-represented in all 3 species, which in humans and cattle is largely attributable to expansion of subgroups TRBV5, 6, 7 and 10 and TRBV6, 9 and 21 respectively; in mice the expansion of subgroups TRBV12 and 13 make a more modest contribution to this over-representation. Much of the expansion of human subgroups TRBV5, 6 and 7 occurred 24–32 MYA [13] and similarly, as described above, in bovines much of the expansion of subgroups TRBV6, 9 and 21 subgroups appears to be very recent. As these expansions have occurred subsequent to primate/artiodactyl divergence (~100MYA) [69], over-representation of phylogenetic groups A and E must have occurred as parallel but independent events in these lineages, raising interesting questions about the evolutionary pressures that shape the functional TRBV repertoire.

In contrast to the wide variation in the organisation of TRBD, TRBJ and TRBC genes in the TRB locus seen in non-mammalian vertebrates [70-74], in mammals the arrangement of tandemly located DJC clusters is well conserved [10,35,36,75,76]. Although most placental species studied have 2, variation in the number of DJC clusters has been observed, with unequal cross-over events between TRBC genes usually invoked as the most likely explanation for this variation [36,77,78]. The results from this study provide the first description of the entire bovine DJC region and confirm that like sheep, cattle have 3 complete DJC clusters [36,37]. Dot-plot and sequence analyses indicate that unequal crossover between the ancestral TRBC1 and TRBC3 genes led to duplication of a region incorporating TRBC1, TRBD3 and TRBJ3 genes, generating the DJC2 cluster. The similarity with the structure of the ovine DJC region suggests that this duplication event occurred prior to ovine/bovine divergence 35.7 MYA [69]. As with duplication of TRBV genes, expansion of TRBD and TRBJ gene numbers has increased the number of genes available to partake in somatic recombination – the 3168 different VDJ permutations possible from the functional genes present in Btau_3.1 is considerably more than that for either humans (42 × 2 × 13 = 1092) or mice (21 × 2 × 11 = 462). Interestingly, the sequence of bovine TRBD1 gene is the first TRBD gene described that doesn't encode a glycine residue (considered integral to the structure of the CDR3β) in all 3 reading frames [79]. However, analysis of cDNA reveals evidence of expression by functional TRB chains of TRBD1in the reading frame that doesn't encode a glycine but have generated a glycine codon by nucleotide editing at the VJ junction (data not shown).

In contrast to TRBV, TRBD and TRBJ genes which encode products that bind to a diverse array of peptide-MHC ligands, TRBC gene products interact with components of the CD3 complex which are non-polymorphic. Consequently, due to structural restrictions TRBC genes are subject to concerted evolutionary pressures with intra-species homogenization through gene conversion evident in both humans and mice [9,80]. Similarly, the bovine TRBC genes were found to encode near identical products, most likely as a result of gene conversion, although in the case of TRBC1 and TRBC2 genes this more probably reflects minimal divergence following duplication.

Comparison with the human and murine sequences shows that non-coding elements that regulate TRB expression, such as the Eβ, promoters and RSs are highly conserved in the bovine. This is consistent with work demonstrating that the critical role of RSs has enforced a high level of evolutionary conservation [70,73,74,81] and that Eβ and PDβ1 sequences are well conserved in eutherian species [36,52]. Although transcriptional factor binding sites are less well conserved in the putative PDβ1 than the Eβ sequence, the Ikaros/Lyf-1 and Ap-1 binding sites of the PDβ1, which are vital in enforcing stage-specific (i.e. Dβ-Jβ prior to Vβ-DβJβ recombination) are conserved [53,82]. Our analysis of putative TRBV promoter elements was restricted to the well described CRE motif [9,10,54]. However, TRBV promoters are complex and expression of TRBV genes whose promoters lack the CRE motif is maintained through the function of other transcriptional factor binding sites [83]. A more detailed analysis of the bovine TRBV promoters would be interesting given the potential influence this may have on shaping the expressed TRBV repertoire [25], but is beyond the scope of the current study.

The portion of the bovine TRB locus described in Btau_3.1 encompasses > 730 Kb of sequence (excluding the regions of undetermined sequence in Chr4.003.105 and Chr4.003.108). Thus, although incomplete, the bovine TRB locus is larger than that of either humans (620 Kb) or mice (700 Kb), mainly as a consequence of the duplications leading to the dramatic expansion of the V genes. In contrast to V genes, duplication of trypsinogen genes within the TRB locus is more limited in the bovine (Figure 2), where only 5 trypsinogen genes were identified, compared to the human and murine where more extensive duplication has lead to the presence of 8 and 20 trypsinogen genes respectively. Despite the differences in duplication events, the organisation of both TR and non-TR genes within and adjacent to the TRB locus exhibits a striking conserved synteny between cattle, humans and mice [9,84]. Indeed, the organisation of genes within the TRB locus and its position relative to adjacent loci is ancient, with marked conserved synteny also demonstrated between eutherian and marsupial mammalian species and, to a large extent, chickens [9,75]. Given the evidence for conserved synteny of TRBV gene organisation despite dissimilar duplication/deletion events between mice, humans and cattle, the results of the analysis completed in this study suggest that several subgroups including TRBV1, 2, 17, 22 and 23, which were not identified in the genome assembly or from cDNA sequences, may have been deleted from the bovine genome (Figure 9). Conservation of synteny would predict that the genomic location of the TRBV27 gene identified from cDNA analysis will be within the region of undetermined sequence in Chr4.003.108 between the TRBV26 and 28 genes (Figure 9).

## Conclusion

The primary purpose of this study was to analyse the sequence data made available from the third bovine genome assembly to gain a better understanding of the bovine TRB gene repertoire and the organization and evolution of the bovine TRB locus. The results of this analysis have shown that: (1) the bovine TRBV genomic repertoire has been dramatically expanded through a complex series of duplication events and although incomplete, is the largest described to date. These duplication events have led to massive expansion in the membership of certain TRBV subgroups, particularly TRBV6, 9 and 21; (2) duplication has generated 3 DJC clusters compared to 2 in humans and mice; (3) the elements that regulate TRB expression and the organisation of genes within and adjacent to the TRB loci exhibit high levels of conservation between humans, mice and cattle. (4) cDNA evidence indicates that a substantial number of TRBV genes, predominantly from the larger subgroups are absent from the current assembly.

Notwithstanding the incomplete assembly of the TRB locus, the results of these analyses clearly demonstrate that cattle possess a phylogenetically diverse repertoire of functional TRB genes that is substantially larger than that described for other species. These findings, together with emerging evidence of similar expansions of gene repertoires for other TR chains in ruminants [85,86] suggest that strong evolutionary pressures have driven a generic enlargement of TR gene numbers, and thus greater potential TR diversity, in the ruminant lineage. Further studies are required to define the full extent of these expansions and to understand their evolutionary basis.

## Methods

### Analysis of the genome

The third bovine genome assembly (Btau_3.1) was retrieved directly from the sequencing centre involved in the Bovine Genome Project [87]. Sequences of bovine TRB genes identified from cDNA analysis [31-34,88] and human and murine TRB genes derived from GenBank sequences [GenBank:U66059–U66061] and [GenBank:AE000663–AE000665] respectively were compared to Btau_3.1 using the BLASTN algorithm tool on the Ensembl website [89]. The locations of the TRB genes identified in Btau_3.1 are provided in Additional file 1.

### Sequence analysis

Basic sequence analysis such as CLUSTALW alignments [90] and translations were conducted using the DNAsis Max v2.7 programme (MiraiBio, Alameda, CA, USA). Comparison of human and genomic bovine TRBV sequences was completed using the IMGT/V-QUEST programme [91] available through the IMGT homepage, [92,93]. Dot-plot analyses were completed using dotter programme [94] and dottup programme from EMBOSS [95]. Genome wide repeats were analysed using the RepeatMasker programme [96]. Sequence logos in Figures 10 and 11 were generated using the Weblogo programme [97].

To be considered functional TRBV gene segment sequences were required to maintain i) splice sites appropriate for RNA editing, ii) open reading frames, which include codons for the conserved cysteine, tryptophan and cysteine residues at positions 23, 41 and 104 (IMGT unique numbering system [43]) respectively and iii) a 23-RS compatible with somatic recombination [98,99].

### Nomenclature

As the sequence of the TRB locus was incomplete, it was not possible to fully implement the IMGT nomenclature system which requires knowledge of the genomic order of genes from the 5' to 3' end of the locus [100]. Genomic bovine TRBV gene subgroups have been named according to the orthologous subgroups in humans and members of subgroups have been given an alphabetic rather than numeric description to avoid confusion with previously published cDNA data [32]. The DJC region of the locus appears complete and so the TRBD, TRBJ and TRBC have been named according to their 5' to 3' order in the genome.

### Phylogenetic analysis

Phylogenetic analysis was performed on the nucleotide sequences of functional TRBV genes (coding sequences) and TRBJ genes (coding sequence + RS) of humans, mice and bovine as identified in Btau_3.1. Neighbour-joining method [101] analysis was performed with the MEGA4 software [102,103], using the uncorrected nucleotide differences (p-distance), which is known to give better results when a large number of sequences which contain a relatively small number of nucleotides are examined [104]. Maximum likelihood analysis was performed under a variety of substitution models (Jukes-Cantor, Kimura 2-parameter, Felenstein 81, Felenstein 84, Tamura-Nei 93 and General Time Reversible) as implemented by the PHYML programme [105,106], using the phylogenetic tree produced by NJ analysis as the primary tree. In each case the reliability of the resulting trees was estimated by the approximate Likelihood Ratio Test (aLRT) method [107].

### Southern blot and cDNA analysis

Southern blots were performed as described in Houston *et al*. [32]. Analysis of bovine TRB cDNA expression included use of the methods described in previous studies [31-33,88]. cDNA sequences for bovine TRBV genes were derived from sequences submitted to public databases: [D90130, AJ006569, AJ006570, AJ006572, AJ006573, AJ006574, AJ006575, AJ006576, D90121, D90123, AJ006583, D90122, D90127, D90124, D90133, L18951, AJ006580, D90131, AJ006579, AJ235264, AJ235265, AJ006578, AJ235266, AJ235267, AJ006577, D90128, D90129, AJ235268, D90125AJ006347, AJ006346 and D90132] and additional un-submitted data that are available on request from the corresponding author.

## Abbreviations

IG: immunolobulin; IMGT: IMGT^®^, the international ImMunoGeneTics information system^®^; RFLP: restriction fragment length polymorphism; TRB: T cell receptor beta chain; TR: T cell receptor; TRBV: β variable gene; TRBJ: β joining gene; TRBD: β diversity gene; TRBC: β constant gene.

## Authors' contributions

TC, JA, AL and WM conceived the study. TC performed the genome analysis, the cDNA and southern blot work, and with JA completed the sequence and phylogenetic analysis. All authors read and approved the manuscript.

## Supplementary Material

Additional file 1 **Table S1 – Location of bovine TRB genes in Btau_3.1**. For each gene the start and stop positions on the scaffold as well as the orientation are shown. Click here for file

Additional file 2 **Table S2 – Summary of the analysis of non-functional TRBV gene segments identified in Btau_3.1**. To be considered functional TRBV gene segment sequences were required to fulfil several conditions as described in the Methods. The table above summarises the lesions identified in the genomic sequences of TRBV genes that are considered to render them non-functional and therefore psuedogenes. TRBV6c, 6g, 6r, 6w and 6aa are open-reading frame (ORF) pseudogenes as their reading frame is maintained, the lesions causing their predicted loss of function resulting either in the loss of a conserved residue or loss of a consensus 23-RS. Click here for file

Additional file 3 **Figure S1 – Analysis of the repeat content in (A) Chr4.003.105, (B) Chr4.003.108_RC and (C) ChrUn.003.1717**. The repeat content is calculated as the percentage (y-axis) of the sequence (x-axis) composed of repeat elements in 10 Kb windows using a rolling 1 Kb step. The positions of TRB genes and interposed/adjacent non-TRB genes, as well as regions of undetermined sequence are shown according to the legend. Abbreviations for gene names used in the figure have been described in Figure 9. Click here for file

## References

[B1] Nikolich-Zugich J, Slifka MK, Messaoudi I (2004). The many important facets of T-cell repertoire diversity. Nat Rev Immunol.

[B2] Arstila TP, Casrouge A, Baron V, Even J, Kanellopoulos J, Kourilsky P (1999). A direct estimate of the human alphabeta T cell receptor diversity. Science.

[B3] Casrouge A, Beaudoing E, Dalle S, Pannetier C, Kanellopoulos J, Kourilsky P (2000). Size estimate of the alpha beta TCR repertoire of naive mouse splenocytes. J Immunol.

[B4] Garboczi DN, Ghosh P, Utz U, Fan QR, Biddison WE, Wiley DC (1996). Structure of the complex between human T-cell receptor, viral peptide and HLA-A2. Nature.

[B5] Garcia KC, Degano M, Stanfield RL, Brunmark A, Jackson MR, Peterson PA, Teyton L, Wilson IA (1996). An alphabeta T cell receptor structure at 2.5 A and its orientation in the TCR-MHC complex. Science.

[B6] Barker PE, Ruddle FH, Royer HD, Acuto O, Reinherz EL (1984). Chromosomal location of human T-cell receptor gene Ti beta. Science.

[B7] Caccia N, Kronenberg M, Saxe D, Haars R, Bruns GA, Goverman J, Malissen M, Willard H, Yoshikai Y, Simon M, Hood L, Mak TW (1984). The T cell receptor beta chain genes are located on chromosome 6 in mice and chromosome 7 in humans. Cell.

[B8] Isobe M, Erikson J, Emanuel BS, Nowell PC, Croce CM (1985). Location of gene for beta subunit of human T-cell receptor at band 7q35, a region prone to rearrangements in T cells. Science.

[B9] Glusman G, Rowen L, Lee I, Boysen C, Roach JC, Smit AF, Wang K, Koop BF, Hood L (2001). Comparative genomics of the human and mouse T cell receptor loci. Immunity.

[B10] Rowen L, Koop BF, Hood L (1996). The complete 685-kilobase DNA sequence of the human beta T cell receptor locus. Science.

[B11] Bosc N, Lefranc MP (2000). The mouse (Mus musculus) T cell receptor beta variable (TRBV), diversity (TRBD) and joining (TRBJ) genes. Exp Clin Immunogenet.

[B12] Folch G, Lefranc MP (2000). The human T cell receptor beta variable (TRBV) genes. Exp Clin Immunogenet.

[B13] Su C, Nei M (2001). Evolutionary dynamics of the T-cell receptor VB gene family as inferred from the human and mouse genomic sequences. Mol Biol Evol.

[B14] McBlane JF, van Gent DC, Ramsden DA, Romeo C, Cuomo CA, Gellert M, Oettinger MA (1995). Cleavage at a V(D)J recombination signal requires only RAG1 and RAG2 proteins and occurs in two steps. Cell.

[B15] Bassing CH, Alt FW, Hughes MM, D'Auteuil M, Wehrly TD, Woodman BB, Gartner F, White JM, Davidson L, Sleckman BP (2000). Recombination signal sequences restrict chromosomal V(D)J recombination beyond the 12/23 rule. Nature.

[B16] Sleckman BP, Bassing CH, Hughes MM, Okada A, D'Auteuil M, Wehrly TD, Woodman BB, Davidson L, Chen J, Alt FW (2000). Mechanisms that direct ordered assembly of T cell receptor beta locus V, D, and J gene segments. Proc Natl Acad Sci USA.

[B17] Tonegawa S (1983). Somatic generation of antibody diversity. Nature.

[B18] Krangel MS (2003). Gene segment selection in V(D)J recombination: accessibility and beyond. Nat Immunol.

[B19] Mostoslavsky R, Alt FW, Bassing CH (2003). Chromatin dynamics and locus accessibility in the immune system. Nat Immunol.

[B20] Schlissel MS (2003). Regulating antigen-receptor gene assembly. Nat Rev Immunol.

[B21] Bories JC, Demengeot J, Davidson L, Alt FW (1996). Gene-targeted deletion and replacement mutations of the T-cell receptor beta-chain enhancer: the role of enhancer elements in controlling V(D)J recombination accessibility. Proc Natl Acad Sci USA.

[B22] Bouvier G, Watrin F, Naspetti M, Verthuy C, Naquet P, Ferrier P (1996). Deletion of the mouse T-cell receptor beta gene enhancer blocks alphabeta T-cell development. Proc Natl Acad Sci USA.

[B23] Mathieu N, Hempel WM, Spicuglia S, Verthuy C, Ferrier P (2000). Chromatin remodeling by the T cell receptor (TCR)-beta gene enhancer during early T cell development: Implications for the control of TCR-beta locus recombination. J Exp Med.

[B24] Oestreich KJ, Cobb RM, Pierce S, Chen J, Ferrier P, Oltz EM (2006). Regulation of TCRbeta gene assembly by a promoter/enhancer holocomplex. Immunity.

[B25] Ryu CJ, Haines BB, Lee HR, Kang YH, Draganov DD, Lee M, Whitehurst CE, Hong HJ, Chen J (2004). The T-cell receptor beta variable gene promoter is required for efficient V beta rearrangement but not allelic exclusion. Mol Cell Biol.

[B26] Sikes ML, Suarez CC, Oltz EM (1999). Regulation of V(D)J recombination by transcriptional promoters. Mol Cell Biol.

[B27] Whitehurst CE, Chattopadhyay S, Chen J (1999). Control of V(D)J recombinational accessibility of the D beta 1 gene segment at the TCR beta locus by a germline promoter. Immunity.

[B28] Baron C, Sachs DH, LeGuern C (2001). A particular TCR beta variable region used by T cells infiltrating kidney transplants. J Immunol.

[B29] Butler JE, Wertz N, Sun J, Sacco RE (2005). Comparison of the expressed porcine Vbeta and Jbeta repertoire of thymocytes and peripheral T cells. Immunology.

[B30] Halsey WA, Palmer BE, DeMartini JC, Howell MD (1999). Analysis of sheep T-cell receptor beta-chain heterogeneity. Immunogenetics.

[B31] Connelley T, MacHugh ND, Burrells A, Morrison WI (2008). Dissection of the clonal composition of bovine alphabeta T cell responses using T cell receptor Vbeta subfamily-specific PCR and heteroduplex analysis. J Immunol Methods.

[B32] Houston EF, Connelley T, Parsons K, MacHugh ND, Morrison WI (2005). Analysis of T-cell receptor BV gene sequences in cattle reveals extensive duplication within the BV9 and BV20 subgroups. Immunogenetics.

[B33] Houston EF, Morrison WI (1999). Identification of seven new TCRBV subfamilies in cattle (Bos taurus). Eur J Immunogenet.

[B34] Tanaka A, Ishiguro N, Shinagawa M (1990). Sequence and diversity of bovine T-cell receptor beta-chain genes. Immunogenetics.

[B35] Watanabe M, Iwasaki Y, Mita Y, Ota S, Yamada S, Shimizu M, Takagaki Y (2007). Porcine T-cell receptor beta-chain: a genomic sequence covering Dbeta1.1 to Cbeta2 gene segments and the diversity of cDNA expressed in piglets including novel alternative splicing products. Mol Immunol.

[B36] Antonacci R, Di Tommaso S, Lanave C, Cribiu EP, Ciccarese S, Massari S (2008). Organization, structure and evolution of 41 kb of genomic DNA spanning the D-J-C region of the sheep TRB locus. Mol Immunol.

[B37] Conrad ML, Pettman R, Whitehead J, McKinnel L, Davis SK, Koop BF (2002). Genomic sequencing of the bovine T cell receptor beta locus. Vet Immunol Immunopathol.

[B38] Antonacci R, Massari S, De Iaco R, Ciccarese S (2001). Assignment of the TRB@ locus encoding the T-cell receptor beta chain to sheep, cattle, goat and river buffalo chromosomes by in situ hybridization. Cytogenet Cell Genet.

[B39] Su C, Jakobsen I, Gu X, Nei M (1999). Diversity and evolution of T-cell receptor variable region genes in mammals and birds. Immunogenetics.

[B40] Connelley T, Burrells A, Machugh ND, Morrison WI (2008). Use of a Pan-Vbeta primer permits the amplification and sequencing of TCRbeta chains expressed by bovine T-cell clones following a single semi-nested PCR reaction. Vet Immunol Immunopathol.

[B41] Arden B, Clark SP, Kabelitz D, Mak TW (1995). Mouse T-cell receptor variable gene segment families. Immunogenetics.

[B42] Arden B, Clark SP, Kabelitz D, Mak TW (1995). Human T-cell receptor variable gene segment families. Immunogenetics.

[B43] Lefranc MP, Pommie C, Ruiz M, Giudicelli V, Foulquier E, Truong L, Thouvenin-Contet V, Lefranc G (2003). IMGT unique numbering for immunoglobulin and T cell receptor variable domains and Ig superfamily V-like domains. Dev Comp Immunol.

[B44] Gudikote JP, Wilkinson MF (2002). T-cell receptor sequences that elicit strong down-regulation of premature termination codon-bearing transcripts. EMBO J.

[B45] Li S, Wilkinson MF (1998). Nonsense surveillance in lymphocytes?. Immunity.

[B46] Di Meo GP, Perucatti A, Schibler L, Incarnato D, Ferrara L, Cribiu EP, Iannuzzi L (2000). Thirteen type I loci from HSA4q, HSA6p, HSA7q and HSA12q were comparatively FISH-mapped in four river buffalo and sheep chromosomes. Cytogenet Cell Genet.

[B47] Carvajal IM, Sen R (2000). Functional analysis of the murine TCR beta-chain gene enhancer. J Immunol.

[B48] Gottschalk LR, Leiden JM (1990). Identification and functional characterization of the human T-cell receptor beta gene transcriptional enhancer: common nuclear proteins interact with the transcriptional regulatory elements of the T-cell receptor alpha and beta genes. Mol Cell Biol.

[B49] Takeda J, Cheng A, Mauxion F, Nelson CA, Newberry RD, Sha WC, Sen R, Loh DY (1990). Functional analysis of the murine T-cell receptor beta enhancer and characteristics of its DNA-binding proteins. Mol Cell Biol.

[B50] Henderson AJ, McDougall S, Leiden J, Calame KL (1994). GATA elements are necessary for the activity and tissue specificity of the T-cell receptor beta-chain transcriptional enhancer. Mol Cell Biol.

[B51] Doty RT, Xia D, Nguyen SP, Hathaway TR, Willerford DM (1999). Promoter element for transcription of unrearranged T-cell receptor beta-chain gene in pro-T cells. Blood.

[B52] Sikes ML, Gomez RJ, Song J, Oltz EM (1998). A developmental stage-specific promoter directs germline transcription of D beta J beta gene segments in precursor T lymphocytes. J Immunol.

[B53] Wang X, Xiao G, Zhang Y, Wen X, Gao X, Okada S, Liu X (2008). Regulation of Tcrb recombination ordering by c-Fos-dependent RAG deposition. Nat Immunol.

[B54] Anderson SJ, Chou HS, Loh DY (1988). A conserved sequence in the T-cell receptor beta-chain promoter region. Proc Natl Acad Sci USA.

[B55] Yang L, Lanier ER, Kraig E (1997). Identification of a novel, spliced variant of CREB that is preferentially expressed in the thymus. J Immunol.

[B56] Funkhouser W, Koop BF, Charmley P, Martindale D, Slightom J, Hood L (1997). Evolution and selection of primate T cell antigen receptor BV8 gene subfamily. Mol Phylogenet Evol.

[B57] Nei M, Gu X, Sitnikova T (1997). Evolution by the birth-and-death process in multigene families of the vertebrate immune system. Proc Natl Acad Sci USA.

[B58] Nei M, Rooney AP (2005). Concerted and birth-and-death evolution of multigene families. Annu Rev Genet.

[B59] Clark SP, Arden B, Kabelitz D, Mak TW (1995). Comparison of human and mouse T-cell receptor variable gene segment subfamilies. Immunogenetics.

[B60] Bosc N, Lefranc MP (2003). The mouse (Mus musculus) T cell receptor alpha (TRA) and delta (TRD) variable genes. Dev Comp Immunol.

[B61] Sitnikova T, Su C (1998). Coevolution of immunoglobulin heavy- and light-chain variable-region gene families. Mol Biol Evol.

[B62] Bradley DG, MacHugh DE, Cunningham P, Loftus RT (1996). Mitochondrial diversity and the origins of African and European cattle. Proc Natl Acad Sci USA.

[B63] Hiendleder S, Lewalski H, Janke A (2008). Complete mitochondrial genomes of Bos taurus and Bos indicus provide new insights into intra-species variation, taxonomy and domestication. Cytogenet Genome Res.

[B64] MacHugh DE, Shriver MD, Loftus RT, Cunningham P, Bradley DG (1997). Microsatellite DNA variation and the evolution, domestication and phylogeography of taurine and zebu cattle (Bos taurus and Bos indicus). Genetics.

[B65] Behlke MA, Chou HS, Huppi K, Loh DY (1986). Murine T-cell receptor mutants with deletions of beta-chain variable region genes. Proc Natl Acad Sci USA.

[B66] Jouvin-Marche E, Trede NS, Bandeira A, Tomas A, Loh DY, Cazenave PA (1989). Different large deletions of T cell receptor V beta genes in natural populations of mice. Eur J Immunol.

[B67] Noonan DJ, Kofler R, Singer PA, Cardenas G, Dixon FJ, Theofilopoulos AN (1986). Delineation of a defect in T cell receptor beta genes of NZW mice predisposed to autoimmunity. J Exp Med.

[B68] Seboun E, Robinson MA, Kindt TJ, Hauser SL (1989). Insertion/deletion-related polymorphisms in the human T cell receptor beta gene complex. J Exp Med.

[B69] Hedges SB, Dudley J, Kumar S (2006). TimeTree: a public knowledge-base of divergence times among organisms. Bioinformatics.

[B70] Chretien I, Marcuz A, Fellah J, Charlemagne J, Du Pasquier L (1997). The T cell receptor beta genes of Xenopus. Eur J Immunol.

[B71] Fellah JS, Durand C, Kerfourn F, Charlemagne J (2001). Complexity of the T cell receptor Cbeta isotypes in the Mexican axolotl: structure and diversity of the VDJCbeta3 and VDJCbeta4 chains. Eur J Immunol.

[B72] Nam BH, Hirono I, Aoki T (2003). The four TCR genes of teleost fish: the cDNA and genomic DNA analysis of Japanese flounder (Paralichthys olivaceus) TCR alpha-, beta-, gamma-, and delta-chains. J Immunol.

[B73] Shigeta A, Sato M, Kawashima T, Horiuchi H, Matsuda H, Furusawa S (2004). Genomic organization of the chicken T-cell receptor beta chain D-J-C region. J Vet Med Sci.

[B74] Zhou H, Bengten E, Miller NW, Clem LW, Wilson M (2003). The T cell receptor beta locus of the channel catfish, Ictalurus punctatus, reveals unique features. J Immunol.

[B75] Parra ZE, Baker ML, Hathaway J, Lopez AM, Trujillo J, Sharp A, Miller RD (2008). Comparative genomic analysis and evolution of the T cell receptor loci in the opossum Monodelphis domestica. BMC Genomics.

[B76] Williams CB, Blankenhorn EP, Byrd KE, Levinson G, Gutman GA (1991). Organization and nucleotide sequence of the rat T cell receptor beta-chain complex. J Immunol.

[B77] Komatsu M, Lamoyi E, Mage RG (1987). Genomic DNA encoding rabbit T cell receptor beta-chains: isotypes and allotypes of C beta. J Immunol.

[B78] Kotzin BL, Barr VL, Palmer E (1985). A large deletion within the T-cell receptor beta-chain gene complex in New Zealand white mice. Science.

[B79] McCormack WT, Tjoelker LW, Stella G, Postema CE, Thompson CB (1991). Chicken T-cell receptor beta-chain diversity: an evolutionarily conserved D beta-encoded glycine turn within the hypervariable CDR3 domain. Proc Natl Acad Sci USA.

[B80] Rudikoff S, Fitch WM, Heller M (1992). Exon-specific gene correction (conversion) during short evolutionary periods: homogenization in a two-gene family encoding the beta-chain constant region of the T-lymphocyte antigen receptor. Mol Biol Evol.

[B81] De Guerra A, Charlemagne J (1997). Genomic organization of the TcR beta-chain diversity (Dbeta) and joining (Jbeta) segments in the rainbow trout: presence of many repeated sequences. Mol Immunol.

[B82] Yang XO, Doty RT, Hicks JS, Willerford DM (2003). Regulation of T-cell receptor D beta 1 promoter by KLF5 through reiterated GC-rich motifs. Blood.

[B83] Halle JP, Haus-Seuffert P, Woltering C, Stelzer G, Meisterernst M (1997). A conserved tissue-specific structure at a human T-cell receptor beta-chain core promoter. Mol Cell Biol.

[B84] Lai E, Concannon P, Hood L (1988). Conserved organization of the human and murine T-cell receptor beta-gene families. Nature.

[B85] Antonacci R, Lanave C, Del Faro L, Vaccarelli G, Ciccarese S, Massari S (2005). Artiodactyl emergence is accompanied by the birth of an extensive pool of diverse germline TRDV1 genes. Immunogenetics.

[B86] Miccoli MC, Antonacci R, Vaccarelli G, Lanave C, Massari S, Cribiu EP, Ciccarese S (2003). Evolution of TRG clusters in cattle and sheep genomes as drawn from the structural analysis of the ovine TRG2@ locus. J Mol Evol.

[B87] Elsik CG, Tellam RL, Worley KC, The Bovine Genome Sequencing and Analysis Consortium (2009). The genome sequence of taurine cattle: a window to ruminant biology and evolution. Science.

[B88] Connelley T (2007). Immunodominance, clonal composition and TCR'beta' repertoire of the bovine CD8+ T-cell response to *Theileria parva*.

[B89] http://www.ensembl.org.

[B90] Thompson JD, Higgins DG, Gibson TJ (1994). CLUSTAL W: improving the sensitivity of progressive multiple sequence alignment through sequence weighting, position-specific gap penalties and weight matrix choice. Nucleic Acids Res.

[B91] Brochet X, Lefranc MP, Giudicelli V (2008). IMGT/V-QUEST: the highly customized and integrated system for IG and TR standardized V-J and V-D-J sequence analysis. Nucleic Acids Res.

[B92] Lefranc MP, Giudicelli V, Kaas Q, Duprat E, Jabado-Michaloud J, Scaviner D, Ginestoux C, Clement O, Chaume D, Lefranc G (2005). IMGT, the international ImMunoGeneTics information system. Nucleic Acids Res.

[B93] http://imgt.cines.fr/.

[B94] Sonnhammer EL, Durbin R (1995). A dot-matrix program with dynamic threshold control suited for genomic DNA and protein sequence analysis. Gene.

[B95] http://emboss.bioinformatics.nl.

[B96] RepeatMasker. http://www.repeatmasker.org.

[B97] Crooks GE, Hon G, Chandonia JM, Brenner SE (2004). WebLogo: a sequence logo generator. Genome Res.

[B98] Akamatsu Y, Tsurushita N, Nagawa F, Matsuoka M, Okazaki K, Imai M, Sakano H (1994). Essential residues in V(D)J recombination signals. J Immunol.

[B99] Hesse JE, Lieber MR, Mizuuchi K, Gellert M (1989). V(D)J recombination: a functional definition of the joining signals. Genes Dev.

[B100] Lefranc MP (2001). Nomenclature of the human T cell receptor genes. Curr Protoc Immunol.

[B101] Saitou N, Nei M (1987). The neighbor-joining method: a new method for reconstructing phylogenetic trees. Mol Biol Evol.

[B102] Tamura K, Dudley J, Nei M, Kumar S (2007). MEGA4: Molecular Evolutionary Genetics Analysis (MEGA) software version 4.0. Mol Biol Evol.

[B103] http://www.megasoftware.net.

[B104] Nei M, Kumar S (2000). Molecular evolution and phylogenetics.

[B105] Guindon S, Gascuel O (2003). A simple, fast, and accurate algorithm to estimate large phylogenies by maximum likelihood. Syst Biol.

[B106] PHYML. http://atgc.lirmm.fr/phyml.

[B107] Anisimova M, Gascuel O (2006). Approximate likelihood-ratio test for branches: A fast, accurate, and powerful alternative. Syst Biol.

