# Supplementary material for: Genomic analysis reveals extensive gene duplication within the bovine TRB locus
Source: BMC Genomics. 2009 Apr 24;10:192. doi: 10.1186/1471-2164-10-192 (PMC2685407; doi:10.1186/1471-2164-10-192)
Supplement: Additional file 1 — Table S1 – Location of bovine TRB genes in Btau_3.1. For each gene the start and stop positions on the scaffold as well as the orientation are shown. [file 1471-2164-10-192-S1.pdf]

Additional file 1

Gene	Scaffold	Start	Stop	Orientation
Xa	Chr4.003.105	145994	146656	+
4a	Chr4.003.105	226843	227337	+
5a	Chr4.003.105	237018	237482	+
9a	Chr4.003.105	243345	243861	+
6a	Chr4.003.105	248247	248716	+
9b	Chr4.003.105	251978	252491	+
6b	Chr4.003.105	259092	259560	+
9c	Chr4.003.105	262969	263482	+
6c	Chr4.003.105	266897	267365	+
9d	Chr4.003.105	269108	269623	+
6d	Chr4.003.105	270816	271284	+
9e	Chr4.003.105	273050	273562	+
6e	Chr4.003.105	275279	275747	+
6f	Chr4.003.105	278411	278879	+
9f	Chr4.003.105	280576	281091	+
9g	Chr4.003.105	285034	285547	+
6g	Chr4.003.105	286573	287041	+
9h	Chr4.003.105	288402	288909	+
6h	Chr4.003.105	290385	290854	+
6i	Chr4.003.105	291780	292247	+
9i	Chr4.003.105	329222	329737	+
6j	Chr4.003.105	330914	331383	+
9j	Chr4.003.105	337343	337856	+
6k	Chr4.003.105	339201	339669	+
6l	Chr4.003.105	341573	342042	+
9k	Chr4.003.105	343960	344458	+
9l	Chr4.003.105	348836	349349	+
6m	Chr4.003.105	350700	351169	+
9m	Chr4.003.105	352908	353404	+
6n	Chr4.003.105	356663	357132	+
9n	Chr4.003.105	358881	359395	+
6o	Chr4.003.105	360754	361223	+
9o	Chr4.003.105	362927	363424	+
6p	Chr4.003.105	364789	365258	+
6q	Chr4.003.105	368067	368535	+
9p	Chr4.003.105	369786	370293	+
6r	Chr4.003.105	371699	372167	+
9q	Chr4.003.105	373909	374405	+
6s	Chr4.003.105	377573	378042	+
9r	Chr4.003.105	379799	380312	+
6t	Chr4.003.105	381671	382140	+
9s	Chr4.003.105	383842	384339	+
6u	Chr4.003.105	385705	386174	+
6v	Chr4.003.105	388982	389451	+
9t	Chr4.003.105	390715	391223	+
6w	Chr4.003.105	392621	393089	+
9u	Chr4.003.105	394819	395317	+
6x	Chr4.003.105	396933	397402	+
9v	Chr4.003.105	399103	399600	+
6y	Chr4.003.105	400966	401435	+
6z	Chr4.003.105	404245	404713	+
9w	Chr4.003.105	405964	406471	+
6aa	Chr4.003.105	407885	408353	+
9x	Chr4.003.105	410083	410580	+
10a	Chr4.003.105	415457	415905	+
5b	Chr4.003.105	417463	417976	+
6ab	Chr4.003.105	419354	419824	+
9y	Chr4.003.105	424218	424726	+
6ac	Chr4.003.105	426149	426617	+
9z	Chr4.003.105	428352	428849	+
6ad	Chr4.003.105	430204	430673	+
9aa	Chr4.003.105	432448	432963	+
6ae	Chr4.003.105	434943	435411	+
7a	Chr4.003.105	437850	438334	+
5c	Chr4.003.105	454229	454735	+
7b	Chr4.003.105	459290	459912	+
13a	Chr4.003.105	464114	464607	+
11a	Chr4.003.105	476682	477177	+
12a	Chr4.003.105	483164	483645	+
12b	Chr4.003.105	498098	498578	+
14a	Chr4.003.105	500600	501067	+
15a	Chr4.003.105	502899	503402	+
16a	Chr4.003.105	507640	508129	+
18a*	Chr4.003.105	518742	519257	+
21a	Chr4.003.105	520450	520950	+

21b	Chr4.003.105	525655	526142	+
18b	Chr4.003.105	530562	531232	+
19a	Chr4.003.105	532562	533075	+
20a	Chr4.003.105	535732	536598	+
21c	Chr4.003.105	542798	543291	+
21d	Chr4.003.105	548219	548687	+
18c	Chr4.003.105	563188	563857	+
19b	Chr4.003.105	565664	566179	+
20b	Chr4.003.105	568813	569692	+
21e	Chr4.003.105	576245	576748	+
21f	Chr4.003.105	582967	583467	+
21g	Chr4.003.105	588175	588662	+
18d	Chr4.003.105	593096	593766	+
19c	Chr4.003.105	595081	595596	+
20c	Chr4.003.105	598246	599098	+

21o	Chr4.003.108	458089	457604	-
21n	Chr4.003.108	451285	450800	-
21m	Chr4.003.108	447956	447467	-
19d	Chr4.003.108	439743	439228	-
20d	Chr4.003.108	436573	435707	-
21i	Chr4.003.108	427726	427233	-
21j	Chr4.003.108	422329	421846	-
21k	Chr4.003.108	418895	418397	-
21l	Chr4.003.108	414100	413610	-
21h	Chr4.003.108	403277	402780	-
19f	Chr4.003.108	398512	398006	-
24a	Chr4.003.108	389368	388866	-
25a	Chr4.003.108	383609	383100	-
26a	Chr4.003.108	381597	381079	-
28a	Chr4.003.108	229148	228437	-
29a	Chr4.003.108	223434	222755	-
29b	Chr4.003.108	220354	219722	-
29c	Chr4.003.108	217870	217233	-
29d	Chr4.003.108	215396	214759	-
29e	Chr4.003.108	212923	212289	-
DB1	Chr4.003.108	192869	192790	-
JB1-1	Chr4.003.108	192205	192130	-
JB1-2	Chr4.003.108	192071	192001	-
JB1-3	Chr4.003.108	191701	191781	-
JB1-4	Chr4.003.108	191202	191124	-
JB1-5	Chr4.003.108	190931	190849	-
JB1-6	Chr4.003.108	190131	190051	-
C1 - EXON 1	Chr4.003.108	187036	186647	-
C1 - EXON 2	Chr4.003.108	185979	185962	-
C1 - EXON 3	Chr4.003.108	185805	185699	-
C1 - EXON 4	Chr4.003.108	185373	185353	-
DB2	Chr4.003.108	182493	182411	-
JB2-1	Chr4.003.108	181835	181758	-
JB2-2	Chr4.003.108	181640	181561	-
JB2-3	Chr4.003.108	181421	181345	-
JB2-4	Chr4.003.108	181303	181228	-
JB2-5	Chr4.003.108	181018	180943	-
C2 - EXON1	Chr4.003.108	177208	176819	-
C2 - EXON3	Chr4.003.108	176353	176247	-
C2 - EXON4	Chr4.003.108	175920	175900	-
DB3	Chr4.003.108	173258	173176	-
JB3-1	Chr4.003.108	172243	172166	-
JB3-2	Chr4.003.108	172049	171970	-
JB3-3	Chr4.003.108	171828	171752	-
JB3-4	Chr4.003.108	171681	171605	-
JB3-5	Chr4.003.108	171562	171489	-
JB3-6	Chr4.003.108	171466	171386	-
JB3-7	Chr4.003.108	171235	171160	-
C3 - EXON1	Chr4.003.108	168502	168113	-
C3 - EXON2	Chr4.003.108	167436	167419	-
C3 - EXON3	Chr4.003.108	167264	167158	-
C3 - EXON4	Chr4.003.108	166884	166864	-
30a	Chr4.003.108	152549	153248	+

5d	ChrUn.003.1717	3838	4303	+
9ai	ChrUn.003.1717	10156	10672	+
6am	ChrUn.003.1717	15055	15523	+
9ah	ChrUn.003.1717	18786	19299	+
6al	ChrUn.003.1717	22719	23187	+
9ag	ChrUn.003.1717	24930	25445	+

6ak	ChrUn.003.1717	26638	27105	+
9af	ChrUn.003.1717	28862	29366	+
6aj	ChrUn.003.1717	31083	31551	+
6ai	ChrUn.003.1717	34216	34684	+
9ae	ChrUn.003.1717	36381	36896	+
9ad	ChrUn.003.1717	40822	41335	+
6ah	ChrUn.003.1717	42680	43148	+
6ag	ChrUn.003.1717	45052	45521	+
9ac	ChrUn.003.1717	47439	47937	+
9ab	ChrUn.003.1717	52315	52828	+
6af	ChrUn.003.1717	54179	54631	+
4b	ChrUn.003.1717	58386	58880	+
19e	ChrUn.003.4367	4493	3978	-
20e	ChrUn.003.4367	1323	457	-
21p	ChrUn.003.4367	13908	13419	-
6an	ChrUn.003.12588	479	948	+

* the 3' end of TRBV18a was not complete in Btau_3.1
