# Supplementary material for: Genomic analysis reveals extensive gene duplication within the bovine TRB locus
Source: BMC Genomics. 2009 Apr 24;10:192. doi: 10.1186/1471-2164-10-192 (PMC2685407; doi:10.1186/1471-2164-10-192)
Supplement: Additional file 2 — Table S2 – Summary of the analysis of non-functional TRBV gene segments identified in Btau_3.1. To be considered functional TRBV gene segment sequences were required to fulfil several conditions as described in the Methods. The table above summarises the lesions identified in the genomic sequences of TRBV genes that are considered to render them non-functional and therefore psuedogenes. TRBV6c, 6g, 6r, 6w and 6aa are open-reading frame (ORF) pseudogenes as their reading frame is maintained, the lesions causing their predicted loss of function resulting either in the loss of a conserved residue or loss of a consensus 23-RS. [file 1471-2164-10-192-S2.doc]

| TRBV gene | No initiation codon | Frameshift in L exon | Stop codon in L exon | Frameshift in V exon | Stop codon in V exon | Defective splice sites | Loss of conserved amino acids | RS defect |
| --- | --- | --- | --- | --- | --- | --- | --- | --- |
| 5a |  |  |  | ● |  | ● |  | ● |
| 5b |  |  |  |  | ● |  |  |  |
| 5d |  |  |  | ● |  | ● |  | ● |
| 6a |  | ● |  |  |  |  |  |  |
| 6c |  |  |  |  |  |  | ● |  |
| 6d |  |  |  | ● |  | ● |  | ● |
| 6e |  |  |  | ● |  | ● |  |  |
| 6f | ● |  |  | ● |  |  |  |  |
| 6g |  |  |  |  |  |  |  | ● |
| 6h |  |  |  | ● |  |  |  |  |
| 6i |  | ● |  |  |  |  |  |  |
| 6j |  |  |  |  | ● |  |  |  |
| 6n |  |  | ● |  |  | ● |  |  |
| 6r |  |  |  |  |  |  |  | ● |
| 6s |  |  |  |  |  | ● |  |  |
| 6w |  |  |  |  |  |  |  | ● |
| 6aa |  |  |  |  |  |  |  | ● |
| 6ab |  |  |  | ● |  |  |  |  |
| 6af |  |  |  | ● |  | ● |  | ● |
| 6ai | ● |  |  | ● |  |  |  |  |
| 6aj |  |  |  |  |  | ● |  |  |
| 6ak |  |  |  | ● |  | ● |  | ● |
| 6al |  |  |  |  |  |  | ● |  |
| 9e |  |  |  | ● |  | ● |  |  |
| 9h |  | ● |  | ● |  |  |  |  |
| 9l |  |  | ● |  |  |  |  |  |
| 9m |  |  |  | ● |  |  |  |  |
| 9p |  | ● |  | ● |  |  |  |  |
| 9q |  |  |  | ● |  |  |  |  |
| 9t |  | ● |  | ● |  |  |  |  |
| 9u |  |  |  | ● |  |  |  |  |
| 9w |  | ● |  | ● |  |  |  |  |
| 9y |  | ● |  | ● |  |  |  |  |
| 9ab |  |  | ● |  |  |  |  |  |
| 9af |  | ● |  | ● |  |  |  |  |
| 10a |  |  |  | ● |  |  |  |  |
| 11a |  |  |  | ● |  |  |  |  |
| 13a | ● | ● | ● | ● |  | ● |  |  |
| 16a | ● |  |  |  |  | ● |  |  |
| 18a |  | ● | ● | ● | ● |  |  |  |
| 18b |  | ● | ● | ● | ● |  |  | ● |
| 18c |  | ● | ● | ● | ● |  |  | ● |
| 18d |  | ● | ● | ● | ● |  |  | ● |
| 19a |  |  |  | ● | ● |  |  |  |
| 19b |  |  | ● |  |  |  |  |  |
| 19c |  |  |  |  | ● |  |  |  |
| 21a |  |  |  |  | ● |  |  |  |
| 21c |  | ● |  |  |  |  |  |  |
| 21e |  | ● | ● |  |  |  |  |  |
| 21i |  | ● |  |  |  |  |  |  |
| 21j |  |  |  | ● |  |  |  |  |
| 21k |  | ● |  |  |  |  |  |  |
| 21n |  |  |  | ● |  |  |  |  |
| 29a |  |  |  | ● |  |  |  |  |
| 29d |  |  |  |  |  | ● |  |  |
