# Supplementary figures and images for: Genomic analysis reveals extensive gene duplication within the bovine TRB locus
Source: BMC Genomics. 2009 Apr 24;10:192. doi: 10.1186/1471-2164-10-192 (PMC2685407; doi:10.1186/1471-2164-10-192)

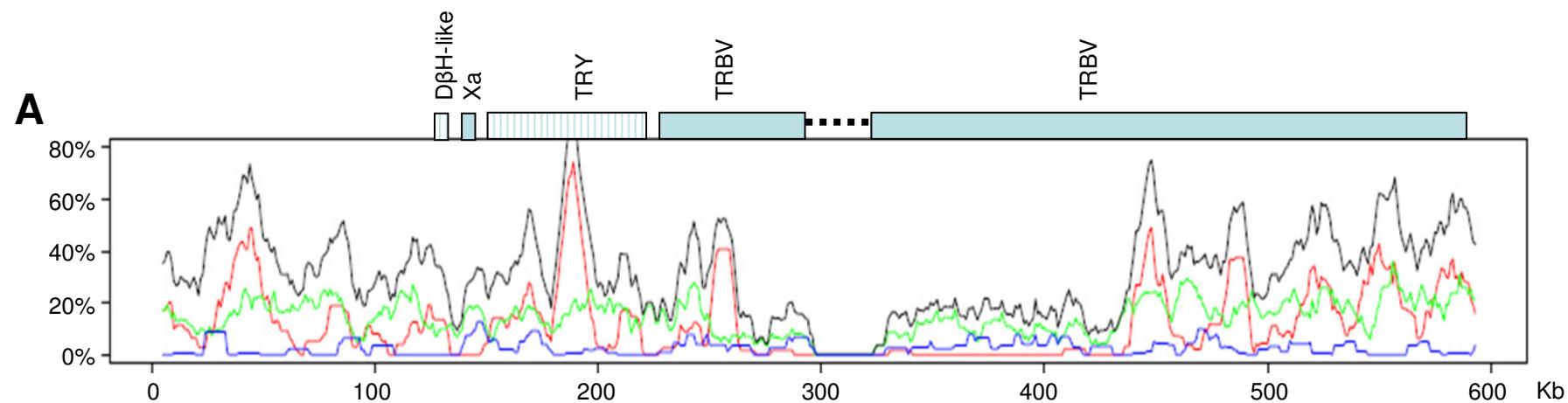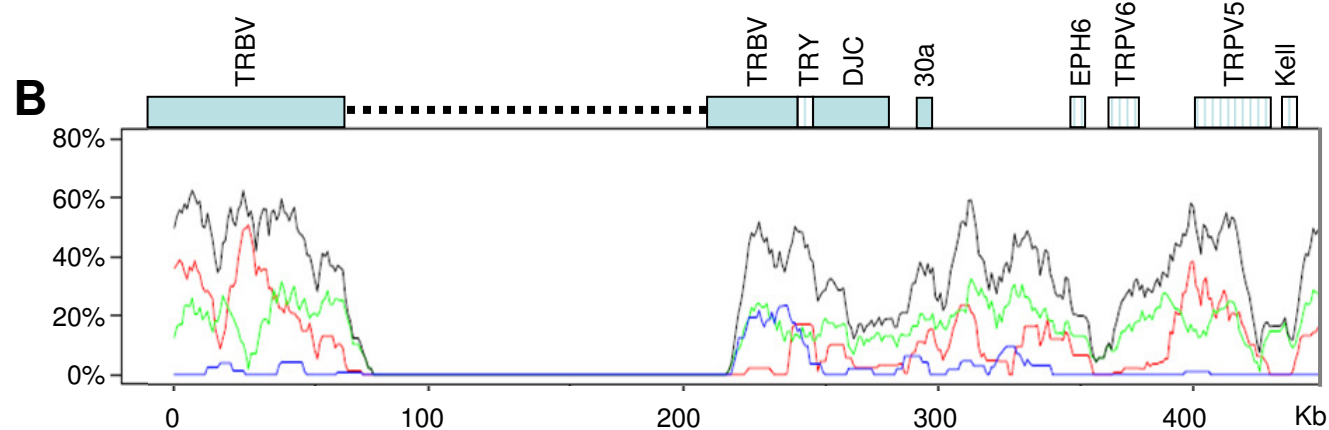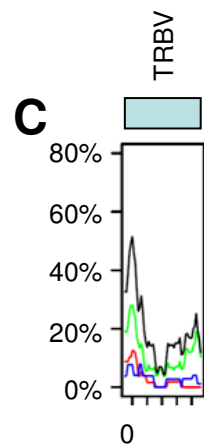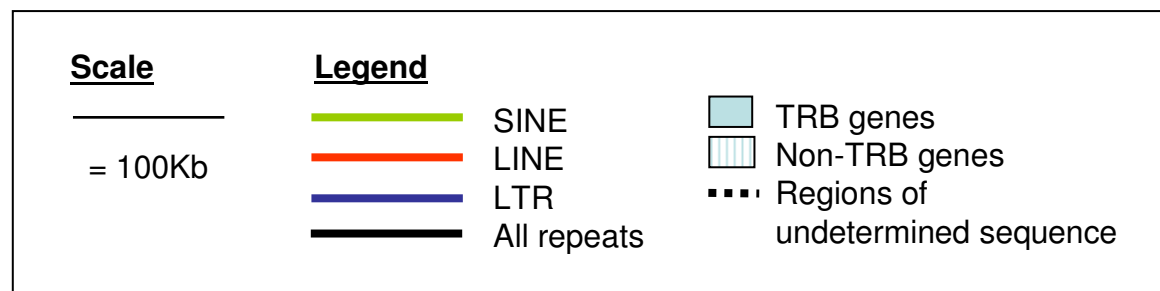

Supplement: Additional file 3 — Figure S1 – Analysis of the repeat content in (A) Chr4.003.105, (B) Chr4.003.108_RC and (C) ChrUn.003.1717. The repeat content is calculated as the percentage (y-axis) of the sequence (x-axis) composed of repeat elements in 10 Kb windows using a rolling 1 Kb step. The positions of TRB genes and interposed/adjacent non-TRB genes, as well as regions of undetermined sequence are shown according to the legend. Abbreviations for gene names used in the figure have been described in Figure 9. [file 1471-2164-10-192-S3.pdf]
